# Association of posterior occlusal support with ultrasonographic characteristics of the masseter muscle: a prospective single-center study

**DOI:** 10.1186/s12903-026-09730-4

**Published:** 2026-09-11

**Authors:** Alperen Tekin, Belde Arsan Özcan, Burcu Sümer, Büşra Erdoğan

**Affiliations:** Department of Oral and Maxillofacial Radiology, Faculty of Dentistry, Istanbul Medeniyet University, Istanbul, Turkey

**Keywords:** Masseter muscle, Ultrasonography, Eichner index, Posterior occlusal support, Derived strain-elastography metric, B-mode image-texture analysis

## Abstract

**Background:**

Posterior occlusal support is associated with masticatory loading, but its relationship with masseter morphometry, a derived strain-elastography metric, and processed B-mode image texture has not been fully characterized. This observational study evaluated the association of posterior occlusal support, classified by the Eichner index, with masseter muscle thickness, estimated masseter volume, a derived strain-elastography metric, and B-mode image-texture fractal dimension in systemically healthy adults using a multimodal ultrasonographic protocol.

**Methods:**

Of 112 enrolled systemically healthy individuals, four did not complete the ultrasonographic protocol; 108 participants (56 female, 52 male; mean age 51.12 ± 12.30 years) were analysed and classified as Eichner group A (*n* = 44), B (*n* = 43), or C (*n* = 21). Bilateral masseter thickness, estimated masseter volume, and a derived strain-elastography metric were assessed at rest and during non-force-standardized maximum voluntary clenching; resting B-mode image-texture fractal dimension was calculated from processed ultrasonographic images. Group comparisons, paired analyses, correlation analyses, and generalized estimating equation models were performed.

**Results:**

Thickness, estimated masseter volume, and the derived strain-elastography metric were higher during clenching than at rest (all *p* < 0.001). Thickness and estimated masseter volume were lower from Eichner A to C under both conditions (all *p* < 0.001). In exploratory analyses, the derived strain-elastography metric was higher in group A than in groups B and C, and resting B-mode image-texture fractal dimension was lower from A to C (*p* < 0.001). Adjusted between-group differences in rest-to-clench change were most consistent for thickness; interactions for estimated masseter volume and the derived strain-elastography metric were model dependent.

**Conclusions:**

Reduced posterior occlusal support was associated with lower masseter thickness, lower estimated masseter volume, lower derived strain-elastography metric values, and smaller rest-to-clench changes, most consistently for thickness. Exploratory findings for B-mode image-texture fractal dimension also differed across Eichner groups but require validation using acquisition-standardized imaging.

**Supplementary Information:**

The online version contains supplementary material available at https://doi.org/10.1186/s12903-026-09730-4.

## Background

The masseter muscle is one of the principal elevator muscles of the mandible and contributes to bite-force generation, chewing, and jaw stabilization. Its morphology is associated with occlusal function, craniofacial pattern, and habitual loading. Previous work has shown relationships of masseter thickness with bite force, electromyographic activity, facial morphology, and oral functional performance; however, an imaging measurement alone should not be regarded as a direct surrogate for clinical function [1–3].

Aging and tooth loss are among the variables associated with this functional environment. Progressive reduction in posterior tooth contacts has been linked to lower masticatory performance, chewing ability, and oral functional reserve, while age-related changes may also affect muscle quantity and quality. Clinical, ultrasonographic, and MRI-based studies have associated tooth loss, number of remaining teeth, functional tooth units, and posterior support with differences in masticatory muscle size, bite force, chewing ability, and oral function. Voxel-based MRI studies have quantified masseter volume and reported age- and sex-related differences and associations with oral functional measures, while population-based MRI has associated masseter cross-sectional area with tooth number [3–9].

More recent MRI-based work has further emphasized that associations between occlusal status and masticatory muscles are not limited to linear dimensions. Diffusion-weighted MRI analyses according to the Eichner index have reported differences in apparent diffusion coefficient values among Eichner groups, suggesting associations with muscle characteristics [10]. In older adults, MRI-derived masseter volume index has also been associated with nutritional and cognitive status [11]. These studies provide important anatomical and biological context, but MRI is not routinely available in dental clinics and does not permit the same real-time assessment during an instructed clenching task as chairside ultrasonography.

In this context, the Eichner index is particularly useful because it reflects the distribution of interarch occlusal contacts in the premolar and molar support zones rather than relying solely on the absolute number of remaining teeth. This distinction is clinically important: patients with similar tooth counts may differ substantially in functional posterior support. The subclass structure of the Eichner system can provide more detailed information on the location and amount of remaining support; however, the main A-B-C groups are often used when sample sizes within individual subclasses are limited. Previous work has demonstrated relationships between Eichner classification, dentition status, masticatory performance, temporomandibular joint-related conditions, and quantitative ultrasound or MRI-derived muscle parameters [10, 12–14].

Ultrasonography is well suited to evaluation of the masseter because it is noninvasive, radiation-free, accessible, repeatable, and capable of real-time bilateral assessment at rest and during clenching. It has been used to investigate muscle dimensions and sonographic appearance in partial edentulism, bruxism, temporomandibular disorders, mandibular asymmetry, and postoperative or rehabilitative follow-up [15–19]. The validity of quantitative ultrasonographic measurements nevertheless depends heavily on acquisition level, probe orientation, pressure, machine settings, and examination standardization [16–19].

Conventional B-mode morphometry does not describe all ultrasound-derived characteristics of skeletal muscle. Strain elastography adds relative information about tissue deformation compared with a reference region, but it does not provide an absolute measurement of intrinsic stiffness. Masseter studies in temporomandibular disorders, bruxism, and healthy adults suggest that protocol-specific elastographic measures may differ across clinical or functional conditions [20–23]. Combining morphometric and relative elastographic information may therefore describe complementary imaging domains, provided that device- and protocol-related limitations are acknowledged.

Fractal analysis may offer an additional quantitative layer for describing processed B-mode image texture. Fractal dimension is sensitive to image acquisition, resolution, segmentation, thresholding, and preprocessing and should not be interpreted as a direct histological or microstructural biomarker. Nevertheless, fractal and related texture descriptors have been used to quantify image complexity in dental and medical imaging, including muscle-related imaging applications [24–27].

Despite these advances, the existing literature has largely examined occlusal status, ultrasonographic morphometry, elastography, MRI-based volume or diffusion metrics, and image texture in separate contexts. The present study therefore evaluated four ultrasound-derived domains within the same cohort: thickness as a conventional morphometric measure, serial-section-based estimated masseter volume as a protocol-specific geometric estimate, a derived strain-elastography metric calculated from the reciprocal of device-displayed ROI ratios, and fractal dimension as a processed B-mode image-texture descriptor. The aim was to examine their associations with Eichner-defined posterior occlusal support and to determine the extent to which these measures conveyed overlapping information [3, 7, 8, 10, 11, 14, 15, 21, 24].

## Methods

### Study design and participants

This prospective single-center observational study was conducted at the Department of Oral and Maxillofacial Radiology, Istanbul Medeniyet University, Istanbul, Turkey. Participant recruitment and data collection were conducted from 5 January to 4 May 2026. Although recruitment and imaging were prospective, posterior occlusal support and the ultrasonographic outcomes were assessed during the study period; the exposure-outcome analyses were therefore cross-sectional and were interpreted as associations. This study is reported in accordance with the STROBE statement for observational studies. The study was conducted in accordance with the Declaration of Helsinki. The study protocol was approved by the Istanbul Medeniyet University Non-Interventional Clinical Research Ethics Committee (ethics approval no: 2026-GOSEK-0132). Written informed consent to participate was obtained from all 112 enrolled individuals before enrollment.

All eligible individuals presenting to the department during the study period were prospectively recruited. Eligibility required age 18 years or older, systemic health, and the ability to classify posterior occlusal support within the Eichner A-B-C framework. Because this was an exploratory single-center observational study using an available prospectively recruited cohort, the sample size was not determined by a formal pre-recruitment power calculation. A total of 112 eligible individuals provided written informed consent and were enrolled. Four did not complete the ultrasonographic examination. Of these non-completers, one belonged to Eichner group A, two to Eichner group B, and one to Eichner group C. One non-completer (Eichner group C) was unable to maintain adequate cooperation, whereas three (one from group A and two from group B) chose to discontinue during the procedure. No ultrasonographic measurements were obtained from these individuals; therefore, no measured observations were discarded and no imputation was required. The final cohort therefore comprised 108 participants, all of whom had complete data and contributed 432 side-condition observations. The number of individuals assessed before enrollment and the reason-specific pre-enrollment exclusions were not systematically recorded.

Participants were classified primarily according to the three main Eichner categories. Group A included participants with occlusal contacts in all four posterior support zones, group B included participants with one to three posterior support zones and/or anterior contacts only, and group C included participants without any occlusal contact. Eichner subclasses were recorded according to the standard A1-A3, B1-B4, and C1-C3 structure for descriptive reporting. Because subclass groups were small and unevenly distributed, the main analyses used the main A-B-C categories. Detailed information on prosthesis type, denture quality or stability, and whether removable prostheses were worn during ultrasonographic examination was not systematically recorded and was therefore not included as an analytical variable. Potential temporomandibular disorders and bruxism were screened by structured interview and routine orofacial examination. Individuals were excluded for a reported diagnosis or treatment of temporomandibular disorder or bruxism, current jaw-muscle or temporomandibular-joint pain, clinically evident parafunctional clenching or grinding, joint sounds or restricted mandibular movement suggestive of active temporomandibular disorder, or marked tooth-wear/parafunctional findings considered compatible with bruxism. No study-specific instrumental bruxism assessment or formal DC/TMD Axis I protocol was applied. Individuals with muscle disease, ongoing orthodontic treatment, a history of facial trauma or surgery, systemic disease, or prior botulinum toxin application affecting the masticatory muscles were also excluded. Biological sex was obtained from the clinical record and categorized as female or male for analysis.

### Ultrasonographic examination

All ultrasonographic examinations were performed using an Esaote MyLab X6 ultrasound system (Esaote S.p.A., Genoa, Italy) operating with Evo 4.1 system software (release 23.00.04, build F100101; 64-bit application architecture) and an SL1543 linear transducer (nominal frequency range, 3.0–13.0 MHz). The same ultrasound system, transducer, participant position, anatomical acquisition levels, and measurement procedures were used throughout. Participants were seated with the head in a neutral position and the Frankfurt horizontal plane parallel to the floor. The B-mode profile, imaging depth, gain, and focal position were adjusted when necessary to obtain adequate visualization; examination-specific settings were not systematically archived. Complete manufacturer- and protocol-specific information, including the system software, transducer, available B-mode profiles, protocol-level depth and gain settings, focal positioning, displayed PRC/PRS/X/M codes, and the limitations of the retained setting records, is provided in Additional file 1: Table S9. Both masseters were examined at rest and during non-force-standardized maximum voluntary clenching.

For the clenching condition, participants were instructed to clench maximally while maintaining the standardized head position, and images were acquired during clenching. Clenching force was not measured or standardized by bite-force or electromyographic monitoring. No force feedback was provided. Information on clench duration, number of trials, rest intervals, a standardized encouragement script, exact mandibular position, confirmation of maximum intercuspation, and whether removable prostheses were worn during imaging was not systematically recorded. Rest-to-clench changes were therefore interpreted as descriptive changes in the ultrasound-derived measurements rather than direct measures of muscle contractile function.

### Masseter muscle thickness assessment

Masseter muscle thickness was assessed bilaterally at rest and during non-force-standardized maximum voluntary clenching. Measurements were obtained on transverse ultrasonographic images from the mid-belly region of the muscle. For each side and condition, 3 repeated measurements were performed in the horizontal plane, and the mean value was used for statistical analysis.

### Estimated masseter volume

Estimated masseter volume was obtained separately for the right and left sides at rest and during clenching. The superior and inferior borders of the masseter were first identified in the longitudinal plane, and the superoinferior muscle length was measured. Because the complete length could not usually be encompassed in one frame, consecutive longitudinal scans were obtained. The inferior end of each acquisition was marked on the skin and used as the reference point for the next acquisition, allowing continuous assessment from the superior to the inferior muscle border. Total muscle length was obtained by summing the consecutive longitudinal segments.

**Fig. 1:**
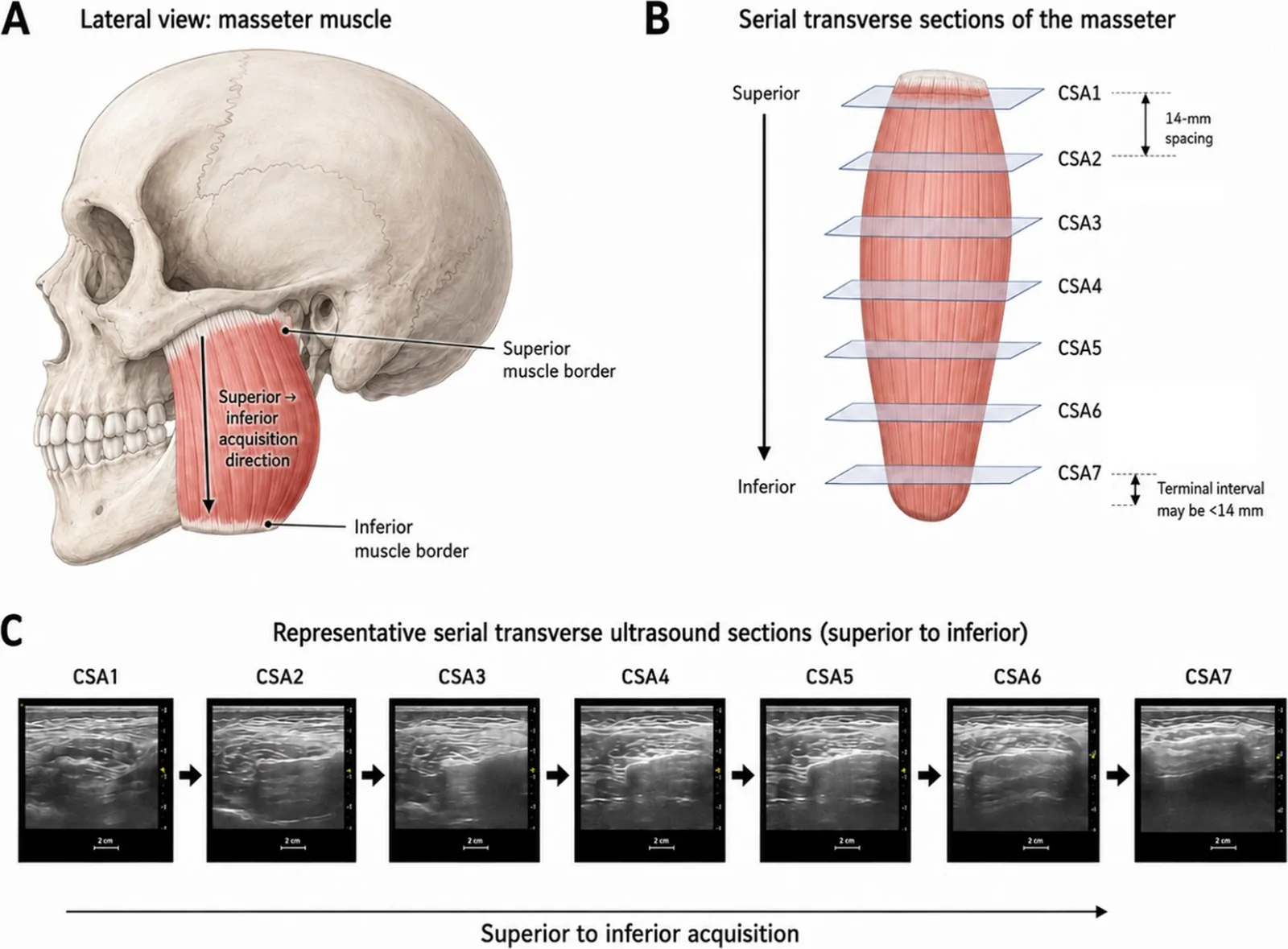
Representative serial transverse-section protocol used for estimated masseter volume calculation. **A** Lateral anatomical view showing the superior-to-inferior acquisition direction. **B** Schematic representation of sequential transverse imaging planes (not to scale). Standard sections were acquired at fixed 14-mm centre-to-centre intervals. If the final interval was shorter than 14 mm, the residual distance between the last two sections was directly measured and used as the final Lᵢ. The number of sections therefore varied according to muscle length, and no zero-area terminal section was assumed. **C** Representative seven-section ultrasound sequence obtained from the same participant. CSA, cross-sectional area

The transducer was subsequently rotated into the transverse plane, and serial cross-sectional images were acquired along the same superoinferior axis. Successive transverse sections were acquired at fixed 14-mm centre-to-centre intervals, derived from the 14-mm short dimension of the SL1543 transducer. Each stored image represented the central imaging plane of the corresponding transducer position. After each acquisition, the inferior edge of the transducer position was marked on the skin, and the superior edge of the transducer was aligned with the preceding mark for the next acquisition. The number of acquired sections varied according to muscle length; seven sections were obtained in the representative sequence shown in Fig. 1.

The first transverse section was acquired immediately inferior to the sonographically identified superior muscle border, at the first level where the complete cross-sectional boundary could be delineated. Serial sections were then obtained inferiorly at fixed 14-mm intervals. If the remaining distance from the last standard section to the inferior muscle border was shorter than 14 mm, that residual distance was directly measured and a final transverse section was acquired adjacent to the inferior border. No zero-area terminal section was assumed.

For the trapezoidal calculation, the actual centre-to-centre distance between each pair of consecutive sections was used. The segment length Lᵢ was 14 mm for every standard interval; when the final interval was shorter than 14 mm, the directly measured residual distance was used as the final Lᵢ. Thus, the standard spacing remained fixed while the number of acquired sections varied with muscle length. Cross-sectional boundaries were manually delineated in Fiji (ImageJ) with the polygon tool, and areas were recorded in mm^2^. The acquisition and calculation procedure is illustrated in Fig. 1.

Estimated masseter volume was calculated using the Average End Area Method (trapezoidal rule) [28]:

$$\mathrm{V}=\sum \left(\mathrm{i}=1\;\mathrm{to}\;\mathrm{n}-1\right)\left[\left({\mathrm{A}}_{\mathrm{i}}+{\mathrm{A}}_{\mathrm{i}+1}\right)/2\right]\times {\mathrm{L}}_{\mathrm{i}}$$

where A_i_ and A_i+1_ are the measured cross-sectional areas of consecutive sections, n is the number of acquired sections, and L_i_ is the actual centre-to-centre distance between those sections. L_i_ was 14 mm for each standard interval, whereas the directly measured residual distance was used for the final interval when it was shorter than 14 mm. The segmental estimates were summed and converted from mm^3^ to cm^3^. This protocol-specific geometric estimate was not validated against MRI-based anatomical volumetry.

Illustrative worked example: for a seven-section sequence with five standard 14-mm intervals and a final measured residual interval of 10 mm, and cross-sectional areas of 349, 406, 428, 406, 388, 382, and 290 mm^2^, respectively, V = 14[(349 + 406)/2 + (406 + 428)/2 + (428 + 406)/2 + (406 + 388)/2 + (388 + 382)/2] + 10[(382 + 290)/2] = 31,269 mm^3^ (31.27 cm^3^). This numerical example is provided only to demonstrate the calculation and does not constitute an additional study result.

### Strain elastography assessment

**Fig. 2:**
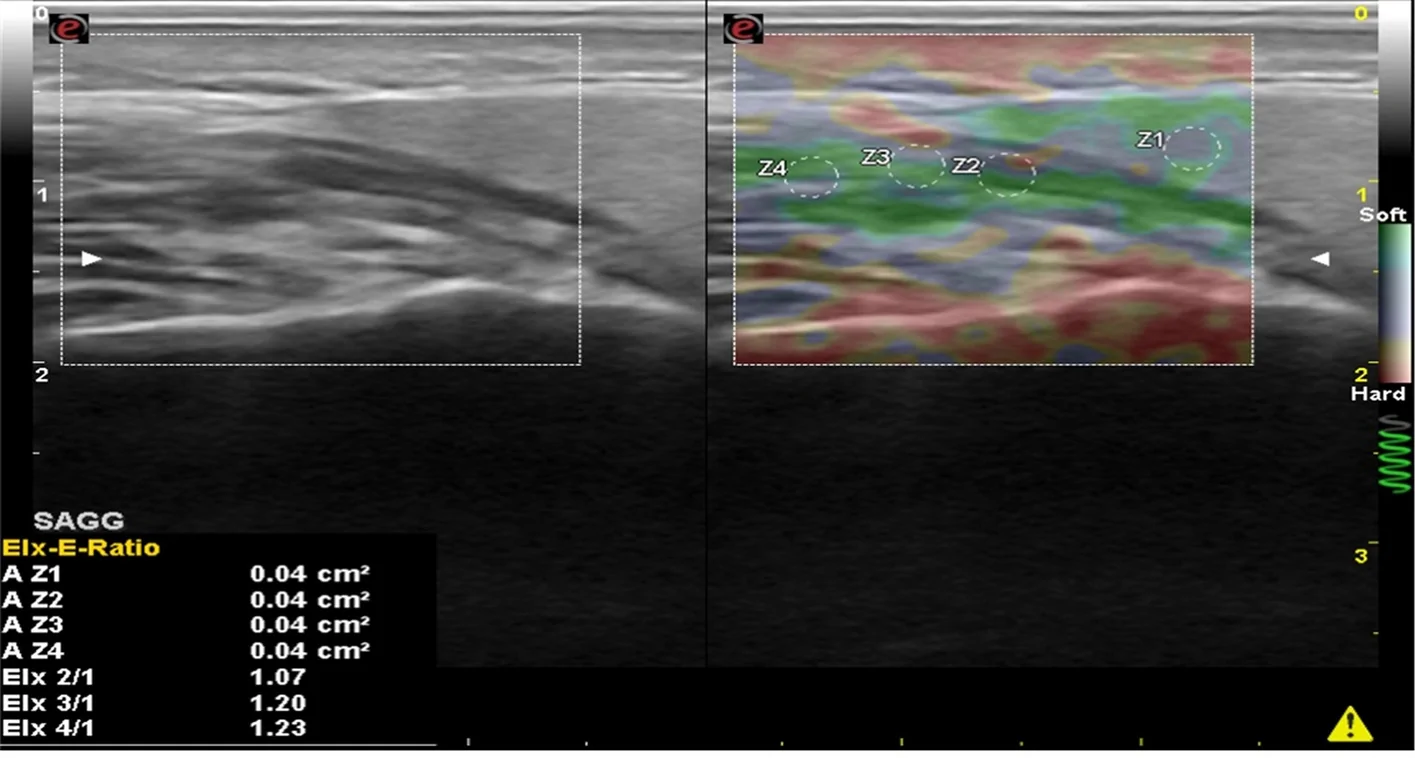
Representative strain-elastography image showing ROI placement. The left panel shows the B-mode image and the right panel the corresponding elastography map. One circular ROI (Z1) was placed in the adjacent parotid gland as the reference tissue, and three circular ROIs (Z2–Z4) were placed within the masseter at comparable depths. Each ROI area was 0.04 cm2. The device-displayed Z2/1, Z3/1, and Z4/1 values were interpreted according to the published ELX convention and averaged; the reciprocal of the mean was used as the protocol-specific derived strain-elastography metric. The metric is reference dependent, and the ratio direction was not independently confirmed by the manufacturer for the specific software configuration

Strain elastography was performed bilaterally at rest and during non-force-standardized maximum voluntary clenching using the same ultrasound system. The ipsilateral parotid gland was used as a reference tissue because strain elastography provides relative, not absolute, deformation information. One circular ROI (Z1) was placed within the parotid gland, and three circular ROIs (Z2-Z4) were placed within the masseter, each standardized to 0.04 cm2. Masseter ROIs were selected at comparable levels and depths relative to the parotid ROI to reduce depth-related variability; representative placement is shown in Fig. 2.

Light rhythmic compression was applied under guidance of the real-time compression-quality indicator. The pre-freeze compression interval was quality-indicator guided rather than fixed to a prespecified duration. The image was frozen when the indicator reached the green range, automatically retaining a 60-s retrospective cine loop. One cine loop was obtained for each side and condition. For the initial measurement, the operator reviewed the cine loop frame by frame and selected the frame showing the highest green level of the helical quality indicator; no additional cine-loop acquisition was obtained for the same side and condition. Thus, each participant contributed four accepted elastography cine-loop acquisitions: right and left sides at rest and during clenching.

Published technical guidance defines the conventional strain ratio as mean strain in a reference tissue divided by mean strain in a target tissue [20]. A published application of the Esaote ElaXto convention placed ROI Z1 in a target thyroid nodule and ROI Z2 in surrounding reference tissue and reported the resulting value as the ELX 2/1 index [29]. These descriptions support, but do not directly confirm for the specific MyLab X6 software configuration used in this study, that the displayed Z2/1 label represents ROI Z2 relative to ROI Z1. Manufacturer confirmation specific to this software configuration was not available.

In the present ROI configuration, Z1 was positioned in the parotid gland and Z2–Z4 within the masseter. On the basis of the published ELX convention, the displayed Z2/1, Z3/1, and Z4/1 values were interpreted as masseter strain relative to parotid strain. The three displayed ratios were averaged, and the reciprocal of this mean was used in the analytical dataset, yielding parotid strain relative to mean masseter strain. Higher values of the derived strain-elastography metric indicate less masseter deformation relative to the parotid during the same compression cycle, which is compatible with greater relative stiffness within this protocol but is not an absolute measurement of intrinsic muscle stiffness. The reciprocal calculation was defined on the participant data-collection sheet before data analysis. The metric has not been externally validated against shear-wave elastography, calibrated stiffness phantoms, or another reference-standard method.

### Semi-automated fractal analysis workflow

**Fig. 3:**
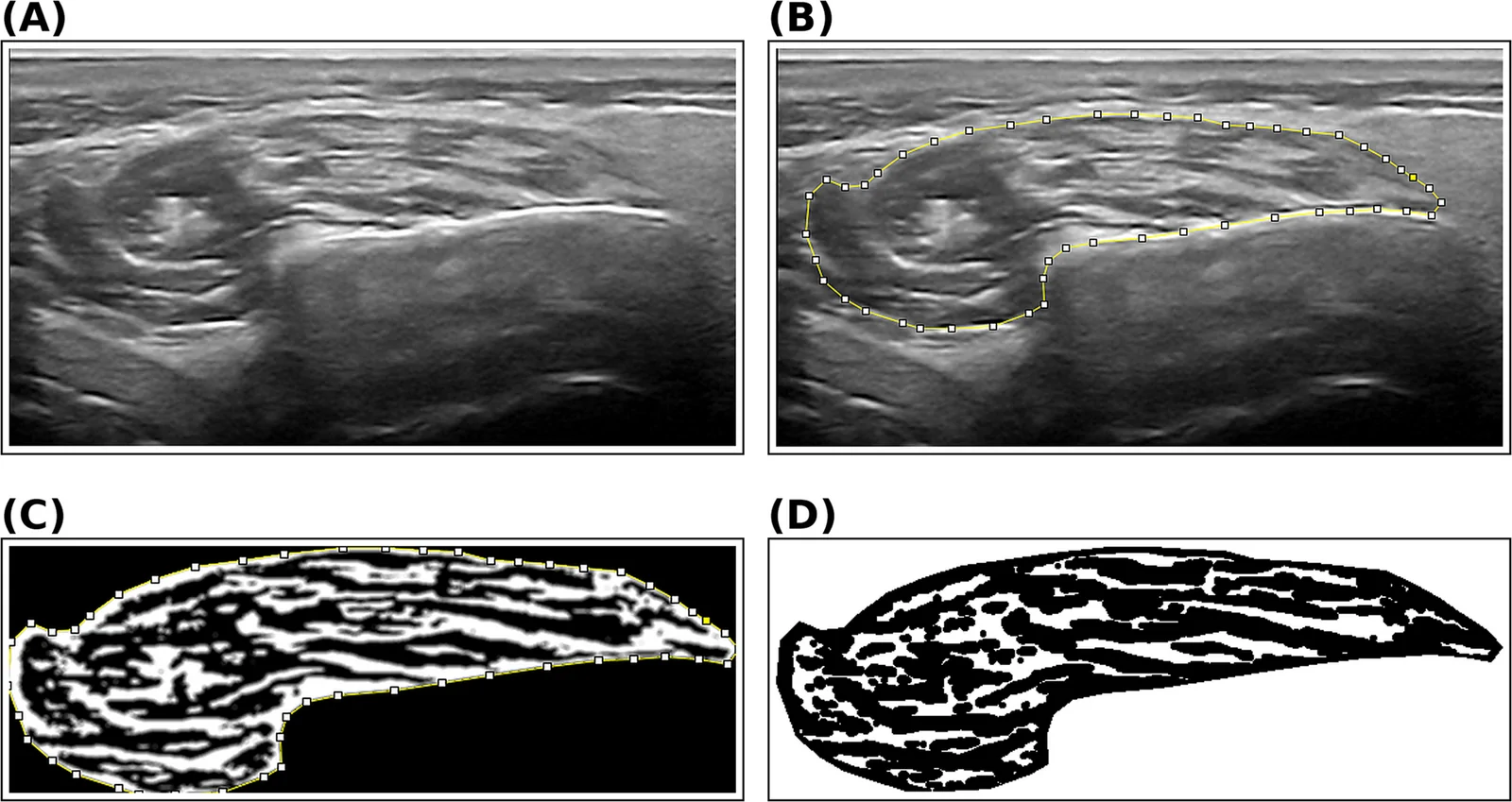
Representative workflow for the B-mode image-texture fractal analysis. **A** Original resting B-mode image. **B** Manual polygon delineation of the masseter ROI in Fiji (ImageJ). **C** Processed ROI after extraction of the segmented masseter region. **D** Final binarized ROI used for box-counting calculation of fractal dimension

Fractal analysis was performed on resting B-mode images of each masseter. The masseter ROI was manually delineated in Fiji/ImageJ (version 1.54p) within the visible muscle borders, excluding the superficial and deep fascia, mandibular bone shadow, and adjacent tissues. Although the ultrasound system, transducer, participant position, and anatomical region were kept consistent, B-mode profile, gain, imaging depth, and focal position were adjusted when necessary for adequate visualization. All ROIs were processed using the same semi-automated macro and identical preprocessing parameters. Fractal dimension was therefore considered a protocol-specific processed B-mode image-texture descriptor rather than a direct histological or microstructural measure. The complete processing workflow is illustrated in Fig. 3.

After segmentation, the area outside the selected masseter ROI was cleared and the ROI was converted to 8-bit grayscale. Background subtraction (rolling = 50) was followed by contrast-limited adaptive histogram equalization (CLAHE; block size = 127, histogram bins = 256, maximum slope = 3). Local Niblack thresholding (radius = 15) was used as an intermediate step to enhance local image contrast before grayscale normalization. A local mean image was generated with a mean filter (radius = 2), and the background-corrected ROI was divided by the corresponding local mean image. The normalized ROI was rescaled twofold using bicubic interpolation and binarized with Otsu thresholding. The final Otsu-binarized image, rather than the intermediate Niblack image, was used for the BoneJ box-counting function. The same macro and plugin settings were applied to all images. The source images were not calibrated to a common physical pixel spacing. Therefore, twofold rescaling was considered an image-processing step rather than physical spatial-resolution normalization. Because operator confirmation was required at one stage, the workflow was classified as semi-automated.

Clinical and dental examinations were performed by BS and BE, and Eichner classification was assigned by BAO. Ultrasound acquisitions were performed by AT. Because the dental examination preceded ultrasonographic imaging and the participants’ dentition was clinically apparent, the ultrasound operator was aware of their dental and posterior occlusal-support status during acquisition. Following acquisition, all images were coded and anonymized before stored-image assessment. Stored-image measurements of masseter thickness, estimated masseter volume, and the derived strain-elastography metric were performed by AT, whereas B-mode image segmentation and B-mode image-texture fractal dimension analysis were performed by BAO. All post-acquisition assessments were conducted using coded images without visible participant identifiers or Eichner-group labels. Therefore, blinding was maintained during stored-image assessment but not during clinical classification or ultrasound acquisition.

A subset of 22 participants, comprising 44 masseter muscles, was randomly selected for intraobserver reliability assessment. Thickness, estimated masseter volume, and B-mode image-texture fractal dimension were remeasured from the stored images by the same observer after a 14-day interval, with the measurement calipers repositioned and the masseter boundaries redelineated. For elastography, a duplicate measurement was obtained during the original examination from the same 60-s cine loop using a separate light-compression cycle, a different frame meeting the same quality criterion, and repositioned Z1–Z4 ROIs. The initial elastography measurement was used in the main analyses and the duplicate only for reliability assessment. To account for the non-independence of the right and left masseters within the same participant, reliability analyses were performed using participant-level bilateral mean values. For each measurement occasion and condition, the right- and left-side values were averaged, and reliability was quantified across the 22 participants using a two-way mixed-effects, absolute-agreement, single-measure intraclass correlation coefficient [ICC(A,1)]. The resulting ICCs therefore represent the reliability of participant-level bilateral mean measurements rather than that of an individual masseter. ICC estimates were reported with 95% confidence intervals. Conventional point-estimate thresholds of 0.50–0.74, 0.75–0.89, and ≥ 0.90 were used as descriptive guides for moderate, good, and excellent reliability, respectively, while interpretation considered both the point estimates and their confidence intervals. Interobserver reliability was not assessed.

### Statistical analysis

Because the Eichner classification reflects global posterior occlusal support rather than side-specific tooth loss, main descriptive and inferential analyses used participant-level bilateral means. Right and left values were averaged within each condition for thickness, estimated masseter volume, and the derived strain-elastography metric. Resting right and left values were averaged for the B-mode image-texture fractal dimension.

Continuous variables were summarized as mean ± standard deviation when approximately normally distributed and as median with interquartile range when distributional assumptions were not met. Categorical variables were presented as counts and percentages. Biological sex, recorded as female or male in the clinical file, was used consistently in analyses. Baseline age differences among Eichner groups were evaluated with one-way analysis of variance, and sex distribution was compared using the chi-square test.

The study protocol did not specify a formal outcome hierarchy. For clarity of reporting and interpretation, masseter thickness was treated as the principal conventional ultrasonographic outcome and estimated masseter volume as a secondary morphometric outcome. The derived strain-elastography metric and B-mode image-texture fractal dimension were treated as exploratory imaging outcomes. Overall rest-to-clench changes were considered secondary descriptive analyses. Condition × Eichner interactions, age- and sex-related analyses, correlations, ordinal models, and additional sensitivity analyses were considered exploratory.

Overall rest-to-clench changes were assessed using paired tests. Paired-samples t-tests were used for bilateral mean thickness and estimated masseter volume, whereas the Wilcoxon signed-rank test was used for the bilateral mean derived strain-elastography metric. Sex-based comparisons used Welch's t-test for thickness and estimated masseter volume and the Mann–Whitney U test for the derived strain-elastography metric and resting B-mode image-texture fractal dimension. Eichner-group comparisons used one-way analysis of variance for thickness and estimated masseter volume and the Kruskal–Wallis test for the derived strain-elastography metric and B-mode image-texture fractal dimension. Significant omnibus tests were followed by Tukey's honestly significant difference test for parametric variables or Holm-adjusted Mann–Whitney U tests for nonparametric variables.

Multiplicity adjustment was applied to post hoc pairwise comparisons within each outcome using Tukey HSD or Holm-adjusted Mann–Whitney U tests, as appropriate. No multiplicity adjustment was applied across the different imaging outcomes or across secondary and exploratory analyses; the corresponding p values were therefore interpreted cautiously and were not considered confirmatory.

Associations between age and imaging-derived variables were assessed using Pearson correlation for approximately normal variables and Spearman rank correlation for nonparametric variables. Correlations among resting bilateral mean thickness, estimated masseter volume, the derived strain-elastography metric, B-mode image-texture fractal dimension, and Eichner severity were examined using Spearman coefficients.

Separate population-averaged generalized estimating equation (GEE) models were fitted for thickness, estimated masseter volume, and the derived strain-elastography metric. Participant identifier was the clustering variable. In the main GEE models, each continuous outcome was modelled with a Gaussian family and identity link so that adjusted coefficients remained interpretable in the original measurement units. An exchangeable working correlation was selected because each participant contributed four correlated side-condition observations without a meaningful serial time ordering. Robust sandwich standard errors were used. Models included condition, age, biological sex, Eichner main group, and side; separate models added condition × Eichner interactions. Adjusted group-specific rest-to-clench changes and 95% confidence intervals were calculated as linear combinations of the condition and interaction coefficients. No outcome was transformed in the main Gaussian-identity models, and the derived strain-elastography metric was analysed in its recorded reciprocal form. Model assessment included inspection of response residuals, standardized residuals, residual-versus-fitted association, and sensitivity to an independence working correlation. Because estimated masseter volume and the derived strain-elastography metric were positive and showed right-tail residual departures, additional exchangeable Gamma-family models with a log link were fitted as distributional sensitivity analyses.

To evaluate whether the principal elastography conclusions depended on the reciprocal transformation, sensitivity analyses were repeated using the original device-displayed mean ratio. These analyses included participant-level rest-to-clench and Eichner-group comparisons and repeated-measures Gaussian-identity and Gamma-log GEE models with the same covariates and clustering structure. Because the original ratio is the inverse scale of the derived metric, directions were expected to reverse; consistency was evaluated on the basis of preserved statistical inference and substantive interpretation. Results are provided in Additional file 1: Table S10.

At the participant level, exploratory proportional-odds ordinal logistic models used Eichner class (A < B < C) as the ordered outcome and the resting bilateral mean imaging variables as standardized predictors. A simultaneous model included all four imaging variables, and two separate-morphometric models entered thickness and estimated masseter volume separately. Because Brant-type threshold-comparison assessments indicated violation of the proportional-odds assumption in all models, pooled ordinal coefficients were treated only as exploratory summaries. Proportional-odds and threshold-specific binary logistic results are reported in Additional file 1: Table S8 and were not used for confirmatory inference or biomarker ranking.

All 108 participants contributed complete right- and left-side resting and clenching data for thickness, estimated masseter volume, and the derived strain-elastography metric, together with bilateral resting B-mode image-texture fractal dimension data. No missing-data imputation was performed. Analyses were performed using IBM SPSS Statistics for Windows, version 26.0 (IBM Corp., Armonk, NY, USA) and Python-based statistical libraries. A two-sided *p* value < 0.05 was considered statistically significant.

## Results

Of the 112 enrolled individuals, four did not complete the ultrasonographic examination. Among these non-completers, one belonged to Eichner group A, two to Eichner group B, and one to Eichner group C. One non-completer (Eichner group C) was unable to maintain adequate cooperation, whereas three (one from group A and two from group B) chose to discontinue during the procedure. No ultrasonographic measurements were obtained from any of the four non-completers. Thus, non-completion was not confined to a single posterior-support category. The final cohort comprised 108 participants (56 female and 52 male; mean age 51.12 ± 12.30 years, range 23–79 years), all of whom were included in the analyses. Each participant contributed four complete side-condition records (right and left sides at rest and during clenching), yielding 432 observations. According to the Eichner main classification, 44 participants were in group A, 43 in group B, and 21 in group C. Participant characteristics are summarized in Table 1. Sex distribution did not differ among groups (χ2 = 0.30, *p* = 0.859), whereas age differed significantly (ANOVA *p* = 0.014), with a post hoc difference between groups A and C (adjusted *p* = 0.013).

**Table 1:** Participant characteristics by Eichner main group

**Group**	**n**	**Age (years)**	**Female, n (%)**	**Male, n (%)**
A	44	47.43 ± 13.28	22 (50.0)	22 (50.0)
B	43	52.26 ± 10.92	22 (51.2)	21 (48.8)
C	21	56.52 ± 10.77	12 (57.1)	9 (42.9)
Total	108	51.12 ± 12.30	56 (51.9)	52 (48.1)

Age was compared with one-way ANOVA (*p* = 0.014); sex was compared with the chi-square test (*p* = 0.859)

Eichner subclass distribution was as follows: A1 *n* = 17, A2 *n* = 14, A3 *n* = 13, B1 *n* = 16, B2 *n* = 14, B3 *n* = 7, B4 *n* = 6, C1 *n* = 7, C2 *n* = 7, and C3 *n* = 7. Because several subclasses, particularly within the lower-support B and C categories, had small sample sizes, formal inferential subclass analyses were not performed. Descriptive subclass summaries are provided in Additional file 1: Table S6.

Across the full cohort, all three ultrasound-derived measurements were higher during clenching than at rest. Bilateral mean thickness increased from 10.40 ± 2.46 mm to 12.64 ± 3.35 mm (*p* < 0.001), bilateral mean estimated masseter volume increased from 14.81 ± 6.23 cm3 to 17.28 ± 7.54 cm3 (*p* < 0.001), and the derived strain-elastography metric increased from a median of 1.028 (IQR, 0.727–1.352) to 1.509 (IQR, 1.170–1.915; *p* < 0.001). These changes describe measurements obtained during a non-force-standardized maximum voluntary clenching task and do not demonstrate a true increase in anatomical muscle volume or intrinsic contractile function. Secondary sex- and age-related analyses are provided in Additional file 1: Tables S1-S2.

**Fig. 4:**
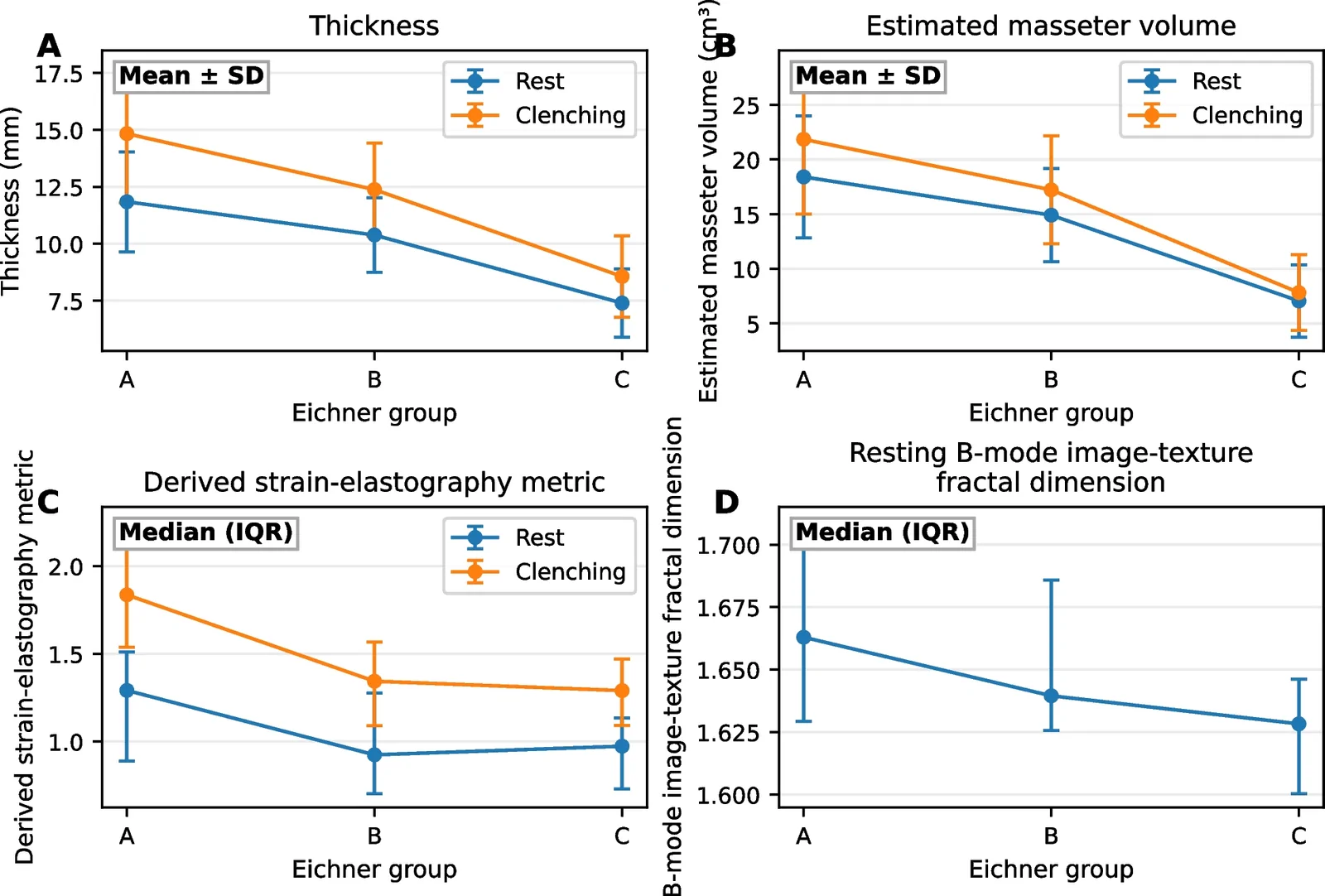
Ultrasound-derived outcomes according to Eichner group. **A** bilateral mean thickness; **B** bilateral mean estimated masseter volume; **C** bilateral mean derived strain-elastography metric; **D** resting B-mode image-texture fractal dimension. Points and error bars indicate mean ± standard deviation for thickness and estimated masseter volume and median with interquartile range for the derived strain-elastography metric and B-mode image-texture fractal dimension. The B-mode image-texture fractal dimension findings shown in panel D are exploratory and intended for hypothesis generation

Eichner main groups differed across the evaluated ultrasound-derived outcomes. Thickness and estimated masseter volume were lower from group A to B to C at rest and during clenching (all *p* < 0.001). The derived strain-elastography metric also differed among groups at rest (*p* = 0.004) and during clenching (*p* < 0.001); values were higher in group A than in groups B and C, whereas B-C differences were not significant. In the exploratory B-mode image-texture analysis, resting fractal dimension was lower from group A to group B to group C (*p* < 0.001) (Table 2; Fig. 4). Full post hoc comparisons are shown in Additional file 1: Table S3.

**Table 2:** Ultrasound-derived outcomes across Eichner main groups

**Outcome**	**Condition**	**A**	**B**	**C**	**Omnibus p**	**Post hoc**
Thickness, mm	Rest	11.84 ± 2.20	10.38 ± 1.64	7.39 ± 1.50	< 0.001	A > B > C
Thickness, mm	Clenching	14.83 ± 3.05	12.38 ± 2.04	8.56 ± 1.79	< 0.001	A > B > C
Estimated masseter volume, cm^3^	Rest	18.41 ± 5.59	14.91 ± 4.25	7.06 ± 3.31	< 0.001	A > B > C
Estimated masseter volume, cm^3^	Clenching	21.85 ± 6.86	17.22 ± 4.94	7.82 ± 3.48	< 0.001	A > B > C
Derived strain-elastography metric	Rest	1.291 (0.889–1.510)	0.924 (0.703–1.276)	0.973 (0.729–1.135)	0.004	A > B = C
Derived strain-elastography metric	Clenching	1.836 (1.538–2.259)	1.344 (1.090–1.567)	1.290 (1.092–1.471)	< 0.001	A > B = C
B-mode image-texture fractal dimension	Rest	1.6630 (1.6294–1.7096)	1.6395 (1.6256–1.6858)	1.6283 (1.6004–1.6462)	< 0.001	A > B > C

Values are mean ± standard deviation for thickness and estimated masseter volume, and median (interquartile range) for the derived strain-elastography metric and B-mode image-texture fractal dimension. Thickness and estimated masseter volume were compared with one-way ANOVA and Tukey HSD; the derived strain-elastography metric and fractal dimension were compared with Kruskal–Wallis and Holm-adjusted Mann–Whitney U tests. The B-mode image-texture fractal dimension findings are exploratory and intended for hypothesis generation

**Fig. 5:**
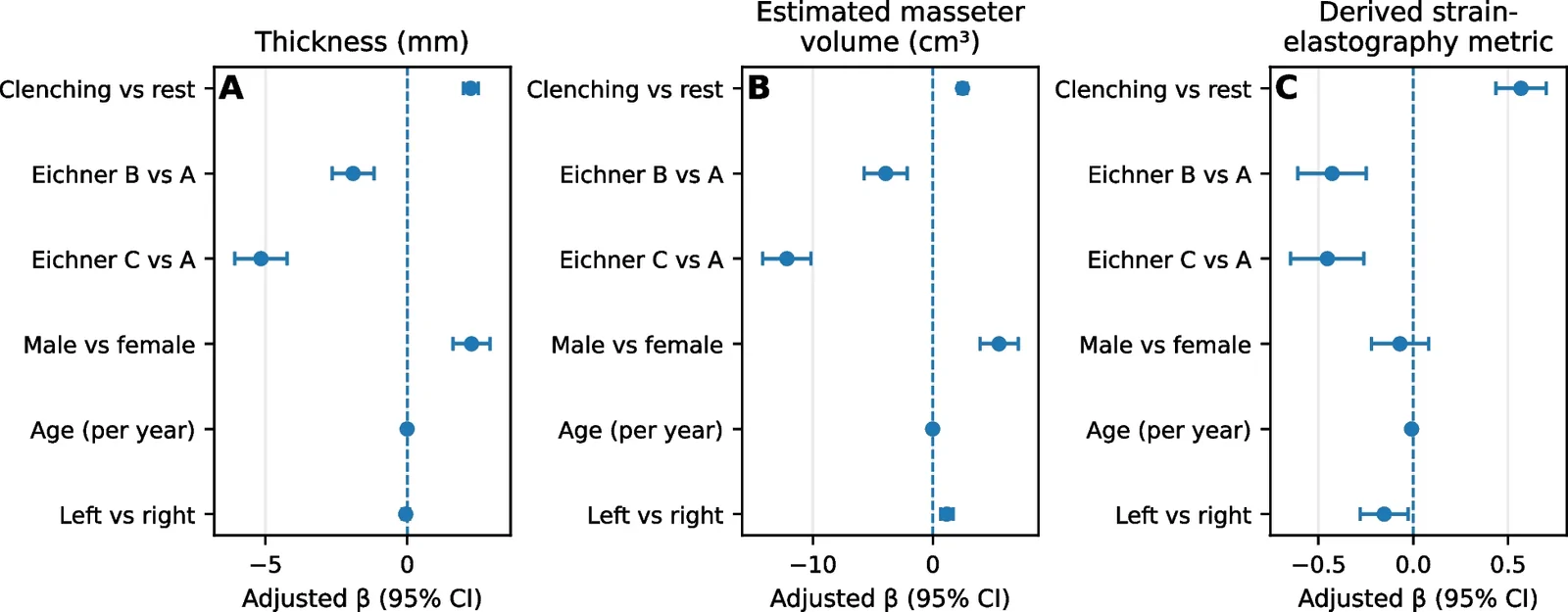
Adjusted coefficients from repeated-measures GEE models. Forest-style plots show beta coefficients with 95% confidence intervals for thickness, estimated masseter volume, and the derived strain-elastography metric

Main Gaussian-identity GEE models showed higher thickness, estimated masseter volume, and derived strain-elastography metric values during clenching than at rest. Compared with Eichner A, groups B and C had lower adjusted values for all three outcomes after accounting for age, biological sex, condition, and side. Male sex was associated with greater thickness and estimated masseter volume but not with the derived strain-elastography metric; age was negatively associated only with the derived strain-elastography metric (Table 3; Fig. 5). In the main-effect models, the exchangeable within-participant correlation estimates were 0.755 for thickness, 0.805 for estimated masseter volume, and 0.106 for the derived strain-elastography metric; the corresponding interaction-model estimates were 0.790, 0.817, and 0.116.

**Table 3:** Main Gaussian-identity population-averaged GEE models for thickness, estimated masseter volume, and the derived strain-elastography metric

**Outcome**	**Predictor**	**β**	**95% CI**	***p***
Thickness, mm	Clenching vs resting	2.240	1.977 to 2.504	< 0.001
Thickness, mm	Eichner B vs A	−1.912	−2.651 to −1.173	< 0.001
Thickness, mm	Eichner C vs A	−5.161	−6.087 to −4.236	< 0.001
Thickness, mm	Male vs female	2.267	1.609 to 2.925	< 0.001
Thickness, mm	Age, per year	−0.004	−0.035 to 0.026	0.789
Estimated masseter volume, cm3	Clenching vs resting	2.471	2.113 to 2.828	< 0.001
Estimated masseter volume, cm3	Eichner B vs A	−3.928	−5.714 to −2.143	< 0.001
Estimated masseter volume, cm3	Eichner C vs A	−12.160	−14.177 to −10.144	< 0.001
Estimated masseter volume, cm3	Male vs female	5.514	3.920 to 7.108	< 0.001
Estimated masseter volume, cm3	Age, per year	−0.015	−0.084 to 0.055	0.675
Derived strain-elastography metric	Clenching vs resting	0.568	0.436 to 0.701	< 0.001
Derived strain-elastography metric	Eichner B vs A	−0.428	−0.609 to −0.248	< 0.001
Derived strain-elastography metric	Eichner C vs A	−0.453	−0.647 to −0.260	< 0.001
Derived strain-elastography metric	Male vs female	−0.070	−0.222 to 0.081	0.362
Derived strain-elastography metric	Age, per year	−0.009	−0.016 to −0.002	0.016

Models used participant as the clustering variable, a Gaussian family, identity link, exchangeable working correlation, and robust sandwich standard errors. They included condition, age, biological sex, Eichner main group, and side. Side coefficients were retained but are not displayed for brevity. Independence-working-correlation sensitivity produced the same coefficients and negligible robust-standard-error differences. Detailed model assessment is provided in Additional file 1: Table S7

**Fig. 6:**
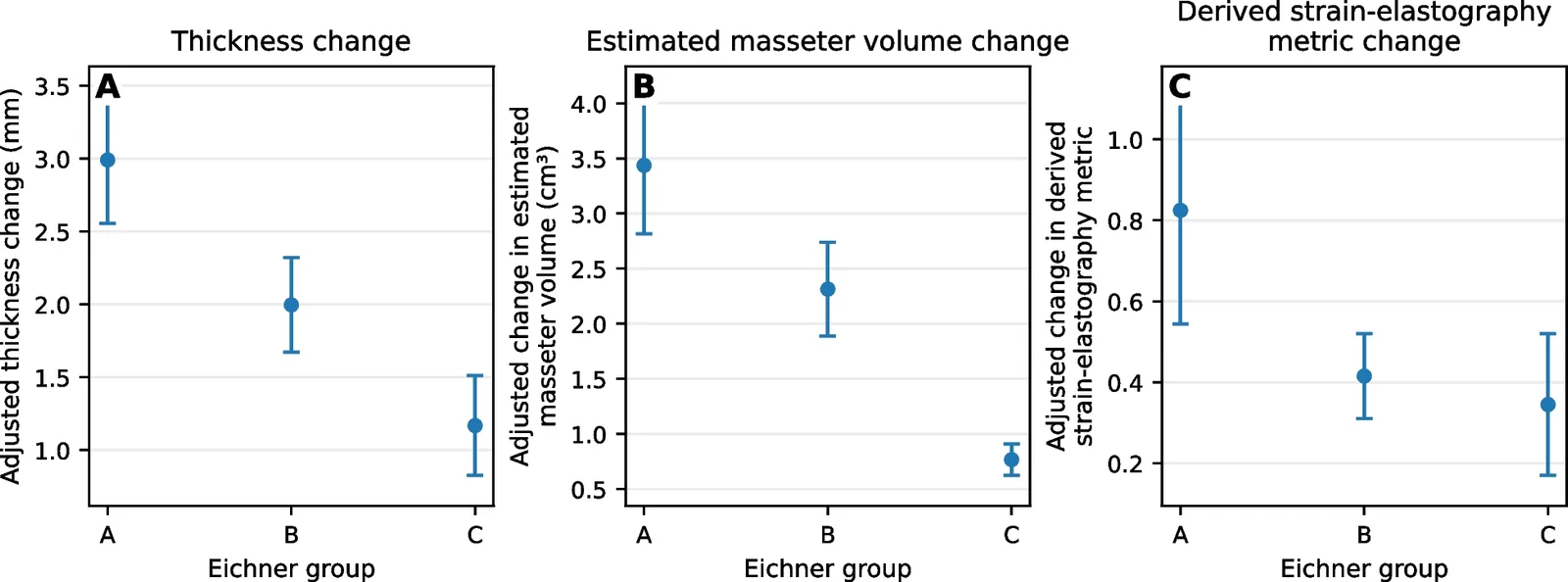
Adjusted group-specific rest-to-clench changes from Gaussian-identity generalized estimating equation models. **A** thickness; **B** estimated masseter volume; **C** derived strain-elastography metric. Points represent adjusted changes and vertical error bars represent 95% confidence intervals. The estimates describe ultrasound-derived changes during non-force-standardized maximum voluntary clenching

Gaussian-identity condition × Eichner models showed smaller adjusted rest-to-clench changes in groups B and C than in group A for thickness, estimated masseter volume, and the derived strain-elastography metric. Adjusted group-specific changes and 95% confidence intervals are shown in Table 4. Distributional sensitivity analyses supported the direction of the main effects. However, in Gamma-log interaction models, the interaction for estimated masseter volume comparing Eichner B with A and both interactions for the derived strain-elastography metric were not statistically significant, whereas the corresponding Eichner C-versus-A interaction for estimated masseter volume was significant. Accordingly, interaction findings were considered most robust for thickness and were interpreted cautiously for estimated masseter volume and the derived strain-elastography metric (Table 4; Fig. 6; Additional file 1: Table S7).

**Table 4:** Adjusted group-specific rest-to-clench changes and condition × Eichner interaction contrasts from Gaussian-identity GEE models

**Outcome**	**Estimate or contrast**	**β/adjusted change**	**95% CI**	***p***
Thickness, mm	Adjusted change: Eichner A	2.991	2.557 to 3.424	< 0.001
Thickness, mm	Adjusted change: Eichner B	1.996	1.671 to 2.322	< 0.001
Thickness, mm	Adjusted change: Eichner C	1.168	0.826 to 1.511	< 0.001
Estimated masseter volume, cm3	Adjusted change: Eichner A	3.437	2.814 to 4.060	< 0.001
Estimated masseter volume, cm3	Adjusted change: Eichner B	2.314	1.888 to 2.740	< 0.001
Estimated masseter volume, cm3	Adjusted change: Eichner C	0.767	0.623 to 0.911	< 0.001
Derived strain-elastography metric	Adjusted change: Eichner A	0.825	0.544 to 1.105	< 0.001
Derived strain-elastography metric	Adjusted change: Eichner B	0.415	0.310 to 0.520	< 0.001
Derived strain-elastography metric	Adjusted change: Eichner C	0.345	0.170 to 0.520	< 0.001
Thickness, mm	Clench × Eichner B vs A	−0.994	−1.537 to −0.452	< 0.001
Thickness, mm	Clench × Eichner C vs A	−1.822	−2.375 to −1.270	< 0.001
Estimated masseter volume, cm3	Clench × Eichner B vs A	−1.123	−1.877 to −0.368	0.004
Estimated masseter volume, cm3	Clench × Eichner C vs A	−2.670	−3.309 to −2.031	< 0.001
Derived strain-elastography metric	Clench × Eichner B vs A	−0.409	−0.709 to −0.110	0.007
Derived strain-elastography metric	Clench × Eichner C vs A	−0.480	−0.811 to −0.149	0.004

Adjusted changes are linear combinations of the condition and interaction coefficients. Negative interaction coefficients indicate a smaller adjusted rest-to-clench change relative to Eichner group A; they do not establish a physiological deficit. Gamma-log sensitivity analyses are reported in Additional file 1: Table S7

Sensitivity analyses using the original device-displayed mean ratio showed the expected inverse pattern. The bilateral median ratio decreased from 1.043 (IQR, 0.786–1.418) at rest to 0.706 (IQR, 0.578–0.941) during clenching (*p* < 0.001). Ratios differed among Eichner groups at rest (*p* = 0.004) and during clenching (*p* < 0.001), with lower values in group A than in groups B and C and no significant B–C difference. In the adjusted Gaussian-identity GEE model, the original ratio was lower during clenching (β = − 0.364, 95% CI − 0.455 to − 0.274; *p* < 0.001) and higher in Eichner B (β = 0.234, 95% CI 0.111 to 0.356; *p* < 0.001) and C (β = 0.228, 95% CI 0.027 to 0.429; *p* = 0.026) than in A. Condition × Eichner interactions were not significant, and Gamma-log sensitivity analyses supported the same substantive interpretation (Additional file 1: Table S10).

**Fig. 7:**
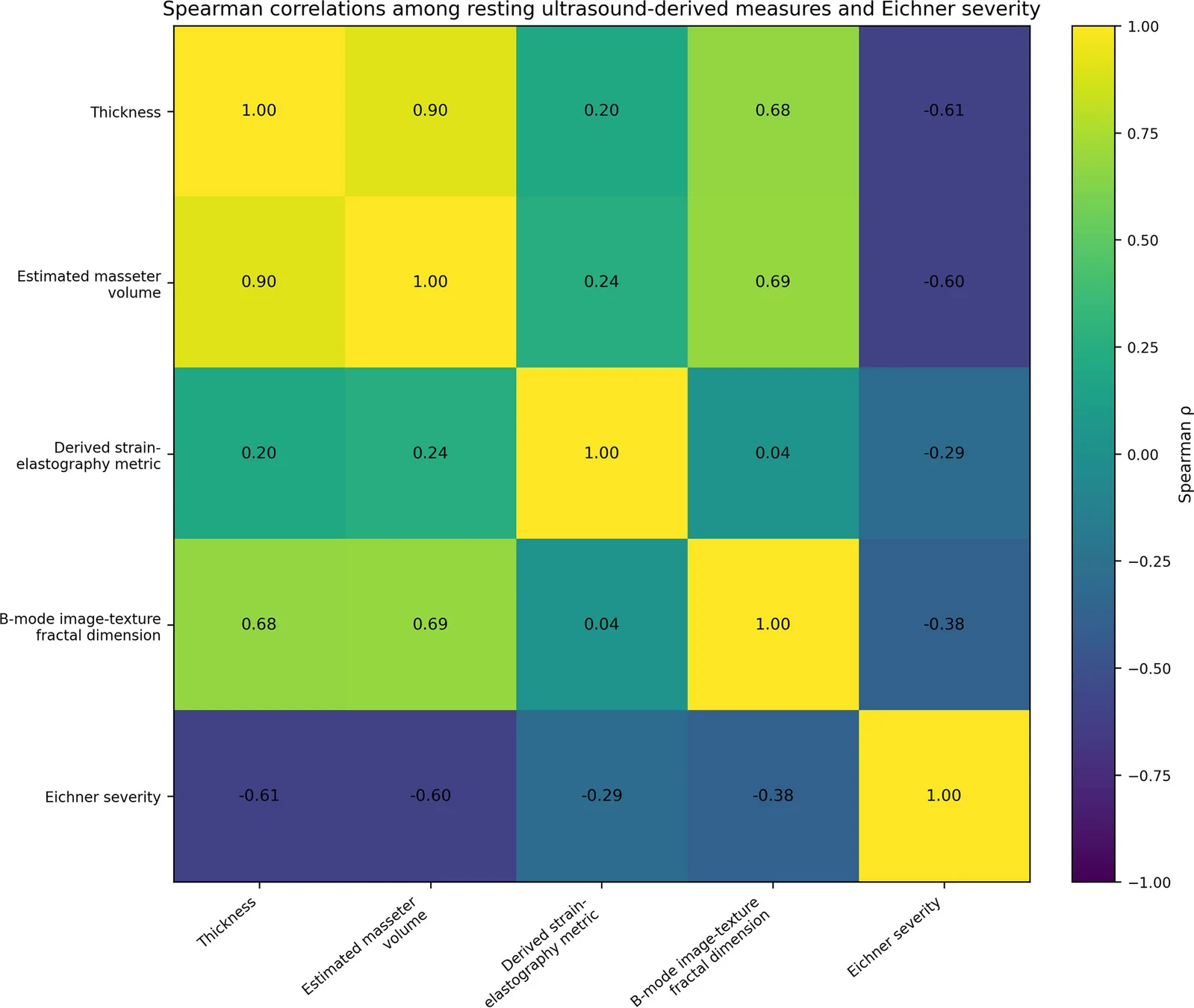
Spearman correlation heatmap of resting ultrasound-derived measures and Eichner severity. Values are Spearman coefficients calculated from participant-level resting bilateral means. All correlation analyses were exploratory, and findings involving B-mode image-texture fractal dimension should be considered hypothesis-generating

Resting side-averaged analyses showed negative correlations of increasing Eichner severity with thickness (*ρ* = −0.612, *p* < 0.001), estimated masseter volume (*ρ* = −0.604, *p* < 0.001), the derived strain-elastography metric (*ρ* = −0.294, *p* = 0.002), and B-mode image-texture fractal dimension (*ρ* = −0.382, *p* < 0.001). Thickness and estimated masseter volume were strongly correlated (*ρ* = 0.904, *p* < 0.001). Fractal dimension correlated with thickness (*ρ* = 0.676, *p* < 0.001) and estimated masseter volume (*ρ* = 0.685, *p* < 0.001), whereas the derived strain-elastography metric showed more modest correlations with morphometric measures (Fig. 7; Additional file 1: Table S4).

In the supplementary participant-level HC3-robust model for the resting derived strain-elastography metric, age was independently associated with lower values (β = −0.008 per year, 95% CI −0.014 to −0.002, *p* = 0.007), whereas Eichner group, biological sex, thickness, and estimated masseter volume were not statistically significant (Additional file 1: Table S5).

Exploratory proportional-odds models did not support a stable ordinal structure because the proportional-odds assumption was violated in the simultaneous and both separate-morphometric models. Accordingly, pooled ordinal coefficients were not used for confirmatory inference or biomarker ranking. Full proportional-odds and threshold-specific results are provided in Additional file 1: Table S8.

Participant-level bilateral-mean ICC(A,1) estimates ranged from 0.85 to 0.95 (Table 5). Resting and clenching thickness showed excellent point estimates, while estimated masseter volume, the derived strain-elastography metric, and resting B-mode image-texture fractal dimension showed good-to-excellent point estimates. The lower confidence limits extended into the moderate-reliability range for some estimated masseter volume and derived strain-elastography measurements. Thickness, estimated masseter volume, and B-mode image-texture fractal dimension reliability reflected stored-image remeasurement after 14 days. Elastography reliability reflected same-session, within-cine-loop repeat-measurement reliability and did not represent participant/probe repositioning or independent reacquisition.

**Table 5:** Intraobserver reliability of participant-level bilateral mean ultrasound-derived measurements

**Variable**	**ICC(A,1)**	**95% confidence interval**
Resting thickness	0.94	0.86–0.97
Clenching thickness	0.95	0.88–0.98
Resting estimated masseter volume	0.85	0.68–0.94
Clenching estimated masseter volume	0.87	0.71–0.94
Resting derived strain-elastography metric	0.87	0.70–0.94
Clenching derived strain-elastography metric	0.85	0.67–0.93
Resting B-mode image-texture fractal dimension	0.90	0.77–0.96

ICCs were calculated from participant-level bilateral mean values for 22 participants using a two-way mixed-effects, absolute-agreement, single-measure model [ICC(A,1)]. Thickness, estimated masseter volume, and B-mode image-texture fractal dimension reflect stored-image remeasurement after 14 days. The derived strain-elastography metric reflects same-session, within-cine-loop repeat-measurement reliability and does not represent full scan-rescan reproducibility

## Discussion

This observational study identified associations between Eichner-defined posterior occlusal support and several ultrasound-derived characteristics of the masseter. The principal finding was a consistent association between reduced posterior occlusal support and lower masseter thickness across the ordered Eichner categories. Estimated masseter volume, considered a secondary morphometric outcome, showed a similar decreasing pattern. Exploratory analyses further showed higher derived strain-elastography metric values in group A than in groups B and C, together with lower resting B-mode image-texture fractal dimension values from group A to group C. Gaussian-identity models showed smaller adjusted rest-to-clench changes in lower-support groups, although distributional sensitivity analyses indicated that interaction findings for estimated masseter volume and the derived strain-elastography metric were less robust than those for thickness. The contribution of this study lies in the evaluation of these protocol-specific ultrasound-derived measures within the same cohort, while the findings should not be interpreted as evidence of causality or physiological impairment.

Previous studies have generally examined occlusal status, conventional ultrasonographic morphometry, elastography, MRI-derived measurements, and image texture in separate settings. The present protocol combined these domains within a single assessment. MRI remains the reference approach for true three-dimensional anatomical volumetry and for evaluation of the deep masticatory muscles, whereas ultrasonography permits bilateral measurements at rest and during an instructed clench and allows morphometric, relative elastographic, and processed image-texture measures to be obtained during the same examination. Accordingly, the present findings should be viewed as exploratory imaging associations rather than evidence of diagnostic or clinical utility.

The lower masseter thickness observed across the main Eichner categories is consistent with previous reports associating reduced posterior support or partial edentulism with smaller masseter dimensions [3, 14, 19]. Rehabilitation studies have also reported changes in masseter thickness after restoration of occlusal support [30], although such longitudinal observations do not establish the explanation for the cross-sectional differences identified here. Furthermore, the ordered group-level pattern should not be interpreted as a uniform biological gradient. Eichner group B includes one to three posterior support zones as well as anterior-only contact, while group C includes clinically diverse patterns of residual dentition and prosthetic rehabilitation. The main categories therefore represent broad patterns of posterior support rather than homogeneous levels of muscular loading.

The estimated masseter volume findings should be interpreted in the context of MRI-based evidence. MRI studies have provided anatomically comprehensive measurements of masseter volume and cross-sectional area and have reported associations with age, sex, tooth loss, oral function, nutritional status, and cognitive status [7–9, 11]. These studies confirm the value of volumetric assessment but also emphasize that MRI is the reference method for detailed anatomical volumetry. The serial-section ultrasound method used in the present study had a more limited purpose: to provide a protocol-specific geometric descriptor broader than a single linear thickness measurement. It was not validated against MRI or another reference-standard volumetric method.

Estimated masseter volume closely paralleled thickness, indicating that the two measures largely represented the same morphometric domain. Potential sources of measurement variation include probe orientation and pressure, reproduction of section levels, movement of skin reference marks, identification of muscle borders, manual segmentation, and changes in muscle geometry during clenching. Consequently, higher estimated masseter volume during clenching may partly reflect changes in cross-sectional shape, probe positioning, compression, and segmentation and should not be interpreted as a true physiological increase in anatomical muscle volume.

The derived strain-elastography metric was higher during clenching than at rest and was higher in Eichner group A than in groups B and C. Previous studies have reported contraction-related or clinical-condition-related differences in masseter elastographic measurements [18, 22, 23]. However, differences in systems, reference tissues, loading procedures, and ratio conventions prevent direct quantitative comparison. Clenching force and electromyographic activation were not standardized. Moreover, the condition × Eichner interactions for the derived strain-elastography metric were statistically significant in the original-scale Gaussian models but not in Gamma-log sensitivity models. The rest-to-clench changes for this metric should therefore be considered model dependent and should not be interpreted as impaired contractility or a specific physiological deficit.

Methodological considerations are particularly important for the elastography findings. Published descriptions support the interpretation that the device ELX 2/1 convention represents ROI2 relative to ROI1 [20, 29], but the ratio direction was not independently confirmed by the manufacturer for the specific MyLab X6 software configuration. With Z1 in the parotid and Z2–Z4 in the masseter, the displayed ratios were therefore interpreted as masseter strain relative to parotid strain, and the reciprocal used in the main analysis represented parotid strain relative to mean masseter strain. Sensitivity analyses using the original device-displayed ratio preserved the statistical evidence for condition and Eichner-group differences with the expected reversal of coefficient direction, indicating that the principal inferences were not created by the reciprocal transformation. Nevertheless, this sensitivity analysis does not independently validate the numerator–denominator convention. Higher derived-metric values remain compatible with less masseter deformation relative to the parotid during the same compression cycle, but the measure is protocol specific and not an absolute index of intrinsic stiffness. Probe compression, ROI position and depth, device processing, and the reference tissue affect strain measurements. The parotid is not mechanically invariant and may vary with age, hydration, glandular composition, medications, and systemic factors. The metric has not been externally validated against shear-wave elastography, calibrated stiffness phantoms, or another reference-standard method.

Resting B-mode image-texture fractal dimension was lower across the Eichner main categories and showed positive correlations with thickness and estimated masseter volume. Directly comparable Eichner-based masseter ultrasonography studies using fractal analysis are lacking. Broader image-analysis literature nevertheless supports the use of fractal descriptors to quantify patterns of image complexity in ultrasound, muscle imaging, and dental imaging [24–26]. These studies provide a rationale for quantitative texture assessment but do not validate a specific histological interpretation of the present values. Accordingly, the observed differences should be described as a protocol-specific processed B-mode image-texture association rather than as evidence of histological simplification, fatty infiltration, or altered muscle microstructure.

The fractal findings are also dependent on the source images and preprocessing workflow. A further consideration is that imaging depth and the selected B-mode profile were individualized according to anatomy, while masseter size and depth differed among participants and may also vary across Eichner categories. Because the source images were not calibrated to a common physical pixel spacing, depth- and profile-related differences in effective pixel scale and speckle appearance may not have been fully independent of Eichner group, even though the settings were not selected on the basis of occlusal status. The positive correlation between fractal dimension and thickness (*ρ* = 0.676) is therefore compatible with both a true within-protocol image-texture association and a partly scale- or acquisition-related effect. Although the same macro standardized segmentation-dependent processing, rescaling, thresholding, and box-counting, it did not standardize the original speckle pattern or physical spatial resolution. Studies evaluating internal sonographic appearance in the masseter have shown that ultrasonography can provide information beyond thickness in other clinical contexts [31, 32], but those studies used different outcomes and do not validate the present fractal workflow. The fractal findings should therefore be regarded as hypothesis-generating pending acquisition-standardized replication with documented pixel calibration, multiobserver assessment, and preferably reference-method validation.

The age- and sex-related findings were more selective than the Eichner-related differences. Males had greater thickness, estimated masseter volume, and resting B-mode image-texture fractal dimension, whereas the derived strain-elastography metric did not differ significantly by sex. This is consistent with previous reports of larger masseter dimensions in males [3, 14]. Older age was associated with several outcomes in bivariate analyses, but in the adjusted GEE models it remained associated only with the derived strain-elastography metric. Evidence that masseter thickness is related to appendicular skeletal-muscle mass and handgrip strength further suggests that general skeletal-muscle status may contribute to masseter measurements [33]. The present data cannot separate age-related, systemic, and occlusal contributions. This pattern was consistent with the supplementary participant-level HC3-robust model, in which age remained independently associated with the resting derived strain-elastography metric while the Eichner-group terms were not statistically significant.

Age differed among Eichner groups, with group C being older than group A. Although age was included in the adjusted models, statistical adjustment reduces but does not eliminate residual confounding, particularly because aging and tooth loss are closely related. Previous ultrasound and MRI studies have linked masseter characteristics with age, tooth loss, nutritional status, and general skeletal-muscle status [3, 9, 11, 33]. Other unmeasured factors included body mass index or body composition, duration of tooth loss, prosthetic status and denture stability, habitual chewing side, dietary consistency, nutritional status, physical activity and sarcopenia-related factors, periodontal condition, and actual clenching force. Accordingly, the observed differences cannot be attributed solely to posterior occlusal support.

The correlation analyses demonstrated substantial overlap between resting thickness and estimated masseter volume. Although exploratory ordinal models were also examined, the proportional-odds assumption was not supported, and their pooled coefficients were therefore not used to compare the relative importance of the imaging measures. The ordinal and threshold-specific findings are reported only as supplementary exploratory analyses.

Statistical significance should also remain distinct from clinical relevance. The study did not measure bite force, chewing efficiency, masticatory performance, patient-reported chewing ability, nutritional outcomes, oral-health-related quality of life, diagnostic accuracy, or treatment response. This distinction is important because the Eichner index has been related to occlusal force and masticatory performance, while tooth loss has been associated with masticatory and nutritional outcomes [6, 13]. The present study therefore cannot determine whether the observed imaging differences represent clinically meaningful functional impairment, support diagnostic use, or permit treatment monitoring. Future studies should standardize or directly measure clenching force, record detailed dentition and prosthetic status, include clinically relevant functional outcomes, and validate the estimated masseter volume and derived strain-elastography measures against appropriate reference methods.

Longitudinal studies are also needed to determine whether these ultrasound-derived measures change after restoration of posterior occlusal support and whether such changes correspond to bite force, masticatory performance, or patient-reported improvement. Previous rehabilitation research reporting masseter changes after restoration of occlusal support provides a rationale for such follow-up [30], but the present cross-sectional findings do not predict responsiveness to prosthetic rehabilitation. Future work should also evaluate acquisition standardization, physical image calibration, interobserver reliability, and complete scan-retest reproducibility.

Several limitations should be acknowledged. Although recruitment and imaging were prospective, posterior occlusal support and the ultrasound-derived outcomes were assessed within the same study period; therefore, causal relationships cannot be established. No formal sample-size calculation was performed before recruitment. Accordingly, the study was not prospectively powered for secondary, interaction, ordinal, or subgroup analyses, and these findings should be interpreted cautiously. Four enrolled participants did not complete the ultrasonographic examination; although non-completion was not confined to a single Eichner category, the small number precluded meaningful assessment of differential attrition. The numbers assessed and excluded before enrollment were not systematically recorded, and selection bias cannot be excluded. Age differed among the Eichner groups, and residual confounding may remain despite statistical adjustment. The main Eichner B and C categories also include heterogeneous patterns of dentition and posterior support. Detailed information on prosthetic rehabilitation, denture quality or stability, duration of tooth loss, body composition, habitual chewing side, nutritional factors, periodontal condition, and actual clenching force was not available. In addition, clenching was not standardized by bite-force or electromyographic monitoring, while TMD and bruxism screening was based on clinical interview and routine examination rather than formal DC/TMD or instrumental assessment. The observed differences therefore cannot be attributed solely to posterior occlusal support or interpreted as direct evidence of impaired muscle function.

The imaging measures were protocol specific. Estimated masseter volume was derived from serial ultrasonographic sections and was not validated against MRI; it may have been influenced by probe orientation and pressure, section positioning, skin-marker displacement, muscle-border delineation, and changes in muscle geometry during clenching. The derived strain-elastography metric was reference dependent and was not externally validated; its ratio direction was inferred from published ELX descriptions rather than independently confirmed by the manufacturer, while the parotid gland may not represent a mechanically invariant reference tissue. B-mode profile, depth, gain, and focal position were adjusted according to individual anatomy, and the source images were not calibrated to a common physical pixel spacing. Differences in B-mode image-texture fractal dimension may therefore partly reflect variations in image acquisition or scale. Some interaction findings for estimated masseter volume and the derived strain-elastography metric varied across the Gaussian and Gamma models. The proportional-odds assumption was not supported for the exploratory ordinal models; therefore, pooled ordinal estimates were not used for confirmatory interpretation or comparison of the imaging measures. Reliability assessment was based on stored-image remeasurement or same-session measurements obtained within the same elastography cine loop rather than a complete repeat ultrasonographic examination. Reliability was assessed using participant-level bilateral mean values, thereby accounting for the dependence between right and left masseters. However, the elastography analysis represented same-session, within-cine-loop repeat-measurement reliability rather than full scan-rescan reproducibility, and interobserver reliability was not evaluated. Accordingly, the elastography reliability estimates may be more favorable than those obtained from a complete test–retest protocol involving participant and transducer repositioning.

These limitations are balanced by prospective recruitment, bilateral assessment, inclusion of all three Eichner main categories, coded stored-image assessment without visible Eichner group labels, and evaluation of morphometric, relative elastographic, and processed image-texture measures within the same cohort. ICC point estimates for stored-image remeasurement reliability and same-session, within-cine-loop repeat-measurement reliability ranged from good to excellent, although confidence intervals for estimated masseter volume and the derived strain-elastography metric indicated greater uncertainty. Overall, the study provides a multidomain description of masseter ultrasound-derived characteristics across broad patterns of posterior occlusal support. The findings remain exploratory and protocol specific and should be interpreted as imaging associations rather than validated functional or clinical biomarkers.

## Conclusion

In conclusion, the most consistent finding was the association of reduced posterior occlusal support with lower masseter thickness. Estimated masseter volume showed a similar secondary morphometric pattern. Exploratory analyses also identified differences in the derived strain-elastography metric and B-mode image-texture fractal dimension. Because no multiplicity adjustment was applied across the different imaging outcomes and several secondary analyses were performed, these findings should be interpreted as protocol-specific imaging associations requiring further validation.

## Supplementary Information

Additional file 1: Supplementary Tables S1–S10. Secondary sex-stratified comparisons, age correlations, full post hoc comparisons, the resting ultrasound-derived measure correlation matrix, HC3-robust regression, descriptive Eichner subclass summaries, GEE model specification and sensitivity analyses, exploratory ordinal and threshold-specific analyses, manufacturer-specific ultrasound configuration, and original device-displayed elastography-ratio sensitivity analyses.

Additional file 2: STROBE checklist. Completed reporting checklist for the observational study.

## Data Availability

The datasets used and/or analysed during the current study are not publicly available because they contain participant-level clinical data, but are available from the corresponding author on reasonable request and subject to applicable ethical and institutional permissions.

## Abbreviations

ANOVA: Analysis of variance
CI: Confidence interval
CLAHE: Contrast-limited adaptive histogram equalization
DC/TMD: Diagnostic Criteria for Temporomandibular Disorders
GEE: Generalized estimating equation
HC3: Heteroskedasticity-consistent type 3
ICC: Intraclass correlation coefficient
IQR: Interquartile range
ROI: Region of interest
SD: Standard deviation
