# Supplementary material for: Association of posterior occlusal support with ultrasonographic characteristics of the masseter muscle: a prospective single-center study
Source: BMC Oral Health. 2026 Sep 11;26:1750. doi: 10.1186/s12903-026-09730-4 (PMC13579981; doi:10.1186/s12903-026-09730-4)
Supplement: Supplementary file 1 [file 12903_2026_9730_MOESM1_ESM.docx]

# Additional file 1: Supplementary Tables S1–S10

Table S1. Sex-stratified comparisons of ultrasound-derived parameters.

| **Variable** | **Male** | **Female** | **Test** | **Statistic** | **p** |
| --- | --- | --- | --- | --- | --- |
| Thickness, rest | 11.48 ± 2.48 | 9.39 ± 1.98 | Welch t-test | 4.824 | <0.001 |
| Thickness, clenching | 14.10 ± 3.58 | 11.28 ± 2.46 | Welch t-test | 4.744 | <0.001 |
| Estimated masseter volume, rest | 17.65 ± 5.93 | 12.17 ± 5.32 | Welch t-test | 5.039 | <0.001 |
| Estimated masseter volume, clenching | 20.63 ± 7.21 | 14.16 ± 6.48 | Welch t-test | 4.892 | <0.001 |
| Derived strain-elastography metric, rest | 1.028 (0.725-1.317) | 1.026 (0.738-1.384) | Mann-Whitney U | 1394.000 | 0.705 |
| Derived strain-elastography metric, clenching | 1.448 (1.170-1.813) | 1.514 (1.204-1.952) | Mann-Whitney U | 1344.000 | 0.493 |
| B-mode image-texture fractal dimension, rest | 1.6653 (1.6486-1.7080) | 1.6280 (1.6197-1.6445) | Mann-Whitney U | 2335.000 | <0.001 |

Thickness and estimated masseter volume are presented as mean ± standard deviation; the derived strain-elastography metric and B-mode image-texture fractal dimension are presented as median (interquartile range). Thickness and estimated masseter volume were compared with the Welch t-test; the derived strain-elastography metric and B-mode image-texture fractal dimension were compared with the Mann-Whitney U test.

Table S2. Correlations between age and ultrasound-derived parameters.

| **Correlation** | **Method** | **r or ρ** | **p** |
| --- | --- | --- | --- |
| Age vs Thickness, rest | Pearson | -0.159 | 0.100 |
| Age vs Thickness, clenching | Pearson | -0.223 | 0.020 |
| Age vs Estimated masseter volume, rest | Pearson | -0.184 | 0.057 |
| Age vs Estimated masseter volume, clenching | Pearson | -0.215 | 0.026 |
| Age vs Derived strain-elastography metric, rest | Spearman | -0.283 | 0.003 |
| Age vs Derived strain-elastography metric, clenching | Spearman | -0.293 | 0.002 |
| Age vs B-mode image-texture fractal dimension, rest | Spearman | -0.065 | 0.505 |

Pearson correlation was used for normally distributed variables and Spearman correlation for nonnormally distributed variables, as shown in the table.

Table S3. Full post hoc pairwise comparisons across Eichner main groups.

| **Outcome** | **Comparison** | **Method** | **Mean difference** | **Adjusted p** |
| --- | --- | --- | --- | --- |
| Thickness, rest | A vs B | Tukey HSD | -1.462 | 0.001 |
| Thickness, rest | A vs C | Tukey HSD | -4.450 | <0.001 |
| Thickness, rest | B vs C | Tukey HSD | -2.989 | <0.001 |
| Thickness, clenching | A vs B | Tukey HSD | -2.456 | <0.001 |
| Thickness, clenching | A vs C | Tukey HSD | -6.272 | <0.001 |
| Thickness, clenching | B vs C | Tukey HSD | -3.817 | <0.001 |
| Estimated masseter volume, rest | A vs B | Tukey HSD | -3.503 | 0.002 |
| Estimated masseter volume, rest | A vs C | Tukey HSD | -11.354 | <0.001 |
| Estimated masseter volume, rest | B vs C | Tukey HSD | -7.852 | <0.001 |
| Estimated masseter volume, clenching | A vs B | Tukey HSD | -4.626 | <0.001 |
| Estimated masseter volume, clenching | A vs C | Tukey HSD | -14.024 | <0.001 |
| Estimated masseter volume, clenching | B vs C | Tukey HSD | -9.399 | <0.001 |
| Derived strain-elastography metric, rest | A vs B | Mann-Whitney U + Holm |  | 0.010 |
| Derived strain-elastography metric, rest | A vs C | Mann-Whitney U + Holm |  | 0.023 |
| Derived strain-elastography metric, rest | B vs C | Mann-Whitney U + Holm |  | 0.977 |
| Derived strain-elastography metric, clenching | A vs B | Mann-Whitney U + Holm |  | <0.001 |
| Derived strain-elastography metric, clenching | A vs C | Mann-Whitney U + Holm |  | <0.001 |
| Derived strain-elastography metric, clenching | B vs C | Mann-Whitney U + Holm |  | 0.502 |
| B-mode image-texture fractal dimension, rest | A vs B | Mann-Whitney U + Holm |  | 0.040 |
| B-mode image-texture fractal dimension, rest | A vs C | Mann-Whitney U + Holm |  | <0.001 |
| B-mode image-texture fractal dimension, rest | B vs C | Mann-Whitney U + Holm |  | 0.028 |

Parametric variables were analyzed with Tukey HSD; nonparametric variables were compared with Mann-Whitney U tests using Holm correction. Adjusted p values are shown.

Table S4. Full Spearman correlation matrix among resting biomarkers and Eichner severity.

| **Variable** | **Thickness** | Estimated masseter volume | Derived strain-elastography metric | B-mode image-texture fractal dimension | **Eichner severity** |
| --- | --- | --- | --- | --- | --- |
| Thickness | 1.000 (<0.001) | 0.904 (<0.001) | 0.201 (0.037) | 0.676 (<0.001) | -0.612 (<0.001) |
| Estimated masseter volume | 0.904 (<0.001) | 1.000 (<0.001) | 0.244 (0.011) | 0.685 (<0.001) | -0.604 (<0.001) |
| Derived strain-elastography metric | 0.201 (0.037) | 0.244 (0.011) | 1.000 (<0.001) | 0.039 (0.688) | -0.294 (0.002) |
| B-mode image-texture fractal dimension | 0.676 (<0.001) | 0.685 (<0.001) | 0.039 (0.688) | 1.000 (<0.001) | -0.382 (<0.001) |
| Eichner severity | -0.612 (<0.001) | -0.604 (<0.001) | -0.294 (0.002) | -0.382 (<0.001) | 1.000 (<0.001) |

The matrix summarizes resting side-averaged biomarkers and Eichner severity. Each cell shows Spearman ρ and p value.

Table S5. HC3-robust multivariable regression model for resting derived strain-elastography metric.

| **Covariate** | **β** | **95% CI** | **p** |
| --- | --- | --- | --- |
| Eichner B vs A | -0.171 | -0.364 to 0.023 | 0.084 |
| Eichner C vs A | -0.037 | -0.352 to 0.277 | 0.816 |
| Male vs female | -0.106 | -0.272 to 0.060 | 0.213 |
| Thickness | 0.000 | -0.075 to 0.076 | 0.990 |
| Estimated masseter volume | 0.016 | -0.009 to 0.042 | 0.215 |
| Age, per year | -0.008 | -0.014 to -0.002 | 0.007 |

A patient-level side-averaged model was fitted for resting derived strain-elastography metric using HC3 heteroskedasticity-consistent standard errors.

**Table S6. Descriptive Eichner subclass distribution and resting ultrasound-derived outcomes.**

Values are descriptive only. Formal inferential subclass analyses were not performed because several subclasses had small sample sizes. Thickness and estimated masseter volume are reported as mean ± standard deviation; the derived strain-elastography metric and B-mode image-texture fractal dimension are reported as median (interquartile range).

| **Main group** | **Subclass** | **n** | **Age, years** | **Resting thickness, mm** | Resting estimated masseter volume, cm³ | Resting derived strain-elastography metric | Resting B-mode image-texture fractal dimension |
| --- | --- | --- | --- | --- | --- | --- | --- |
| A | A1 | 17 | 41.65 ± 11.87 | 12.20 ± 2.08 | 19.34 ± 6.00 | 1.304 (1.109-1.533) | 1.6645 (1.6294-1.7088) |
| A | A2 | 14 | 50.43 ± 13.26 | 12.14 ± 2.54 | 18.97 ± 5.84 | 1.214 (0.749-1.339) | 1.6659 (1.6405-1.7124) |
| A | A3 | 13 | 51.77 ± 13.22 | 11.06 ± 1.88 | 16.60 ± 4.67 | 1.103 (0.721-1.633) | 1.6494 (1.6278-1.6981) |
| B | B1 | 16 | 47.69 ± 10.45 | 10.26 ± 1.92 | 14.71 ± 4.52 | 0.983 (0.819-1.345) | 1.6395 (1.6361-1.6895) |
| B | B2 | 14 | 53.71 ± 8.29 | 9.99 ± 1.63 | 13.98 ± 4.07 | 0.749 (0.692-1.136) | 1.6352 (1.6126-1.6669) |
| B | B3 | 7 | 57.57 ± 13.54 | 10.76 ± 1.40 | 16.11 ± 3.59 | 0.761 (0.678-1.272) | 1.6529 (1.6221-1.6733) |
| B | B4 | 6 | 54.83 ± 12.45 | 11.18 ± 0.97 | 16.21 ± 5.05 | 0.783 (0.692-0.926) | 1.6419 (1.6280-1.6590) |
| C | C1 | 7 | 60.00 ± 9.27 | 7.27 ± 1.34 | 8.02 ± 4.56 | 1.129 (0.968-1.258) | 1.6237 (1.6207-1.6297) |
| C | C2 | 7 | 54.86 ± 11.16 | 8.43 ± 1.43 | 8.25 ± 2.56 | 0.973 (0.762-1.250) | 1.5720 (1.5542-1.6420) |
| C | C3 | 7 | 54.71 ± 12.46 | 6.48 ± 1.17 | 4.91 ± 1.07 | 0.659 (0.540-0.938) | 1.6443 (1.6279-1.6505) |

Table S7. GEE model specification, residual assessment, and distributional sensitivity analyses.

| **Outcome** | **Primary model** | **Exchangeable α (main; interaction)** | **Residual assessment** | **Sensitivity findings** |
| --- | --- | --- | --- | --- |
| Thickness | Gaussian family; identity link; robust sandwich SE | 0.755; 0.790 | Skewness 0.232; excess kurtosis 0.069; approximately symmetric | Independence working correlation produced identical coefficients and negligible robust-SE changes. |
| Estimated masseter volume | Gaussian family; identity link; robust sandwich SE | 0.805; 0.817 | Skewness 0.864; excess kurtosis 2.017; moderate right-tail departure | Gamma/log main effects were consistent. Gamma interaction: B vs A p=0.129; C vs A p<0.001 (Gamma α 0.857; 0.859). |
| Derived strain-elastography metric | Gaussian family; identity link; robust sandwich SE | 0.106; 0.116 | Skewness 1.957; excess kurtosis 6.929; marked right-tail departure | Gamma/log main effects were consistent. Gamma interaction: B vs A p=0.102; C vs A p=0.089 (Gamma α 0.149; 0.153). |

Primary models used a Gaussian family, identity link, exchangeable working correlation, participant clustering, and robust sandwich standard errors. Exchangeable correlation estimates are reported separately for the main-effect and interaction models. An independence working-correlation sensitivity analysis produced identical coefficients with negligible changes in robust standard errors. Because estimated masseter volume and the derived strain-elastography metric showed right-tail residual departures, Gamma-family log-link sensitivity models were fitted. Main-effect direction and significance were stable, but some non-thickness interaction terms were model dependent and were interpreted cautiously.

Table S8. Exploratory ordinal and threshold-specific logistic regression analyses for Eichner classification.

| **Model** | **Predictor** | **OR per 1 SD** | **95% CI** | **p** |
| --- | --- | --- | --- | --- |
| Simultaneous | Resting thickness | 0.323 | 0.107 to 0.975 | 0.045 |
| Simultaneous | Estimated masseter volume | 0.519 | 0.180 to 1.498 | 0.225 |
| Simultaneous | Derived strain-elastography metric | 0.598 | 0.383 to 0.932 | 0.023 |
| Simultaneous | B-mode image-texture fractal dimension | 0.927 | 0.564 to 1.525 | 0.765 |
| Thickness sensitivity | Resting thickness | 0.183 | 0.096 to 0.352 | <0.001 |
| Thickness sensitivity | Derived strain-elastography metric | 0.570 | 0.367 to 0.886 | 0.012 |
| Thickness sensitivity | B-mode image-texture fractal dimension | 0.883 | 0.540 to 1.441 | 0.618 |
| Estimated masseter volume sensitivity | Estimated masseter volume | 0.206 | 0.112 to 0.381 | <0.001 |
| Estimated masseter volume sensitivity | Derived strain-elastography metric | 0.621 | 0.404 to 0.955 | 0.030 |
| Estimated masseter volume sensitivity | B-mode image-texture fractal dimension | 0.861 | 0.528 to 1.402 | 0.547 |

**Threshold-specific binary logistic analyses**

| **Model** | **Cumulative threshold** | **Predictor** | **OR per 1 SD** | **95% CI** | **p** |
| --- | --- | --- | --- | --- | --- |
| Simultaneous | B/C vs A | Resting thickness | 0.386 | 0.117 to 1.270 | 0.117 |
| Simultaneous | B/C vs A | Estimated masseter volume | 0.797 | 0.261 to 2.440 | 0.692 |
| Simultaneous | B/C vs A | Derived strain-elastography metric | 0.524 | 0.320 to 0.857 | 0.010 |
| Simultaneous | B/C vs A | B-mode image-texture fractal dimension | 0.868 | 0.473 to 1.594 | 0.648 |
| Simultaneous | C vs A/B | Resting thickness | 0.282 | 0.026 to 3.075 | 0.299 |
| Simultaneous | C vs A/B | Estimated masseter volume | 0.074 | 0.005 to 1.064 | 0.056 |
| Simultaneous | C vs A/B | Derived strain-elastography metric | 0.887 | 0.385 to 2.044 | 0.779 |
| Simultaneous | C vs A/B | B-mode image-texture fractal dimension | 0.748 | 0.315 to 1.780 | 0.512 |
| Thickness sensitivity | B/C vs A | Resting thickness | 0.318 | 0.155 to 0.654 | 0.002 |
| Thickness sensitivity | B/C vs A | Derived strain-elastography metric | 0.515 | 0.317 to 0.839 | 0.008 |
| Thickness sensitivity | B/C vs A | B-mode image-texture fractal dimension | 0.849 | 0.466 to 1.548 | 0.594 |
| Thickness sensitivity | C vs A/B | Resting thickness | 0.031 | 0.006 to 0.167 | <0.001 |
| Thickness sensitivity | C vs A/B | Derived strain-elastography metric | 0.644 | 0.299 to 1.383 | 0.259 |
| Thickness sensitivity | C vs A/B | B-mode image-texture fractal dimension | 0.679 | 0.301 to 1.530 | 0.350 |
| Estimated masseter volume sensitivity | B/C vs A | Estimated masseter volume | 0.380 | 0.196 to 0.737 | 0.004 |
| Estimated masseter volume sensitivity | B/C vs A | Derived strain-elastography metric | 0.536 | 0.331 to 0.866 | 0.011 |
| Estimated masseter volume sensitivity | B/C vs A | B-mode image-texture fractal dimension | 0.776 | 0.425 to 1.419 | 0.411 |
| Estimated masseter volume sensitivity | C vs A/B | Estimated masseter volume | 0.022 | 0.004 to 0.133 | <0.001 |
| Estimated masseter volume sensitivity | C vs A/B | Derived strain-elastography metric | 1.020 | 0.462 to 2.252 | 0.961 |
| Estimated masseter volume sensitivity | C vs A/B | B-mode image-texture fractal dimension | 0.770 | 0.319 to 1.862 | 0.562 |

Joint Brant-type threshold-comparison tests suggested non-proportional odds for the simultaneous model (chi-square=20.14, df=4, p<0.001), the thickness sensitivity model (chi-square=14.56, df=3, p=0.002), and the estimated masseter volume sensitivity model (chi-square=16.12, df=3, p=0.001). Therefore, the ordinal coefficients are exploratory summaries only. Threshold-specific binary logistic estimates are reported in the preceding table.

Table S9. Ultrasound system, acquisition, and B-mode image-processing configuration.

| **Parameter** | **Reported configuration** | **Qualification** |
| --- | --- | --- |
| Ultrasound system | Esaote MyLab X6 (Esaote S.p.A., Genoa, Italy) | The same ultrasound system was used for all examinations. |
| System software | Evo 4.1; release 23.00.04; build F100101; 64-bit application architecture | Configuration displayed on the system used during data collection. |
| Transducer | SL1543 linear transducer; nominal bandwidth 3.0-13.0 MHz; retained documentation lists 14 x 47 mm dimensions | The same transducer was used throughout. The 14-mm short dimension guided sequential transverse positioning for the estimated masseter-volume protocol. |
| Participant position | Seated; head in a neutral position; Frankfurt horizontal plane parallel to the floor | The same positioning protocol was used throughout. |
| Functional conditions | Bilateral imaging at rest and during non-force-standardized maximum voluntary clenching | No bite-force transducer or electromyographic normalization was used. |
| Available B-mode profiles | RES-H, GEN-M, GEN-L, and PEN-H | These are B-mode frequency-profile labels rather than examination-preset names. Profile selection was individualized; the exact profile and transmitted frequency were not archived for each examination. |
| Imaging depth | Protocol range 22-52 mm | Adjusted when necessary to obtain complete masseter visualization. Examination-specific values were not systematically archived. |
| Gain | 40% usual starting setting | Adjusted when necessary according to image quality. Examination-specific values were not systematically archived. |
| Focal position | Positioned at the level of the masseter muscle | Adjusted according to muscle depth; the exact numerical focal depth was not archived for each examination. |
| PRC display code | 12/1/2/1 | MyLab display sequence for dynamic range / dynamic compression / density / gray map. Reported from the retained configuration; examination-specific changes were not separately archived. |
| PRS display code | 2 | Persistence display code reported from the retained configuration. |
| X/M display code | C2/2 | Displayed XView/CrystaLine and MView code. The code is retained as displayed because the exact algorithm mapping was not independently verified for the software configuration used. |
| Smoothing | No separate B-mode smoothing value was displayed or systematically recorded | No numerical smoothing setting can therefore be reported. |
| Examination-specific setting records | No complete participant-level export or log was available for profile, exact transmitted frequency, depth, gain, focal position, PRC, PRS, or X/M | Groupwise setting distributions across Eichner categories cannot be reconstructed. The table combines verified protocol-level ranges/starting settings with values displayed on the retained system configuration. |

*Note. PRC, PRS, and X/M are machine-display codes, not preset names. RES-H, GEN-M, GEN-L, and PEN-H are B-mode frequency-profile labels and do not provide a separately archived numeric transmitted frequency. Protocol-level ranges and starting settings are distinguished from representative display codes and from unavailable participant-level setting records.*

**Table S10. Sensitivity analyses using the original device-displayed mean elastography ratio.**

Because the original device-displayed ratio is the inverse scale of the derived reciprocal metric, directions are expected to reverse. Consistency was judged by preservation of statistical inference and substantive interpretation rather than by identical coefficient signs.

**Panel A. Participant-level descriptive and nonparametric analyses.**

| **Analysis** | **Group/condition** | **Median** | **Q1** | **Q3** | **p** | **Interpretation** |
| --- | --- | --- | --- | --- | --- | --- |
| Paired rest-to-clench | Rest | 1.043 | 0.786 | 1.418 | <0.001 | Original ratio decreased during clenching. |
| Paired rest-to-clench | Clenching | 0.706 | 0.578 | 0.941 |  |  |
| Eichner comparison, rest | A | 0.893 | 0.704 | 1.149 | 0.004 | A < B = C after Holm-adjusted pairwise comparisons. |
| Eichner comparison, rest | B | 1.213 | 0.885 | 1.448 |  |  |
| Eichner comparison, rest | C | 1.070 | 1.028 | 1.385 |  |  |
| Eichner comparison, clenching | A | 0.580 | 0.503 | 0.679 | <0.001 | A < B = C after Holm-adjusted pairwise comparisons. |
| Eichner comparison, clenching | B | 0.823 | 0.668 | 0.968 |  |  |
| Eichner comparison, clenching | C | 0.785 | 0.745 | 0.960 |  |  |

**Panel B. Gaussian-identity repeated-measures GEE main-effect model.**

| **Predictor** | **β** | **95% CI** | **p** | **Interpretation** |
| --- | --- | --- | --- | --- |
| Clenching vs resting | −0.364 | −0.455 to −0.274 | <0.001 | Expected inverse direction relative to the derived metric. |
| Eichner B vs A | 0.234 | 0.111 to 0.356 | <0.001 | Expected inverse direction relative to the derived metric. |
| Eichner C vs A | 0.228 | 0.027 to 0.429 | 0.026 | Expected inverse direction relative to the derived metric. |
| Male vs female | 0.033 | −0.085 to 0.151 | 0.585 | Not significant. |
| Age, per year | 0.006 | 0.002 to 0.011 | 0.005 | Higher original ratio with age. |

**Panel C. Gaussian-identity condition × Eichner interaction model.**

| **Predictor** | **β** | **95% CI** | **p** | **Interpretation** |
| --- | --- | --- | --- | --- |
| Clenching vs resting in Eichner A | −0.346 | −0.464 to −0.229 | <0.001 | Original ratio decreased during clenching. |
| Clench × Eichner B vs A | 0.003 | −0.183 to 0.189 | 0.976 | No evidence of differential change. |
| Clench × Eichner C vs A | −0.099 | −0.382 to 0.183 | 0.491 | No evidence of differential change. |

Gamma-log GEE sensitivity analyses preserved the direction and statistical significance of the condition and Eichner main effects; condition × Eichner interactions remained non-significant. These findings support the substantive conclusions on the expected inverse scale but do not independently validate the manufacturer-specific numerator–denominator convention.
