# Supplementary material for: Association of posterior occlusal support with ultrasonographic characteristics of the masseter muscle: a prospective single-center study
Source: BMC Oral Health. 2026 Sep 11;26:1750. doi: 10.1186/s12903-026-09730-4 (PMC13579981; doi:10.1186/s12903-026-09730-4)
Supplement: Supplementary file 2 [file 12903_2026_9730_MOESM2_ESM.docx]

**Additional file 2: STROBE checklist**

Completed reporting checklist for the prospective observational study submitted to BMC Oral Health. Locations are provided by manuscript section to avoid dependence on page/line numbering after journal conversion.

| **Item no.** | **STROBE item** | **Manuscript location** | **How addressed** |
| --- | --- | --- | --- |
| 1 | Title and abstract | Title / Abstract | Study design, setting, enrolled-to-analysed participant flow, outcomes, and principal findings are stated. |
| 2 | Background/rationale | Background | Scientific background and rationale are described, including the gap in integrated ultrasonographic assessment. |
| 3 | Objectives | Background | The study objective is stated at the end of the Background. |
| 4 | Study design | Methods - Study design and participants | The prospective single-center observational design and cross-sectional exposure-outcome interpretation are specified. |
| 5 | Setting | Methods - Study design and participants | Department, institution, recruitment context, and the exact recruitment/data-collection period (5 January to 4 May 2026) are described in the Methods. |
| 6a | Participants - eligibility and selection | Methods - Study design and participants; Results | All eligible individuals presenting during the study period were prospectively recruited. Eligibility and exclusion criteria and consent from 112 enrolled individuals are reported. Four did not complete ultrasonography: one Eichner C participant could not maintain adequate cooperation, whereas three participants (A n=1, B n=2) chose to discontinue. The final cohort comprised 108 participants. |
| 6b | Participants - matching criteria | Not applicable | No matched design was used. |
| 7 | Variables | Methods - Ultrasonographic examination / Statistical analysis | Thickness, estimated volume, derived strain-elastography metric, fractal dimension, Eichner group, age, sex, side, and condition are defined. |
| 8 | Data sources/measurement | Methods - Ultrasonographic examination; Figs. 1-3 | Ultrasound device/transducer, positioning, acquisition settings, measurement procedures, ROI workflow, elastography configuration, and fractal-processing workflow are described. |
| 9 | Bias | Methods / Discussion | Standardized device/operator workflow, blinded stored-image measurement, outcome-specific reliability assessment, individualized acquisition settings, unavailable participant-level parameter logs, and relevant limitations are reported. |
| 10 | Study size | Methods - Study design and participants | The available prospectively recruited cohort, 112 enrolled and 108 analysed participants, and absence of a formal pre-recruitment sample-size calculation are stated. |
| 11 | Quantitative variables | Methods - Statistical analysis | Averaging strategy, resting/clenching variables, coding of Eichner severity, and odds-ratio scaling are described. |
| 12a-b, 12d-e | Statistical methods | Methods - Statistical analysis and reliability | Group comparisons, paired tests, correlations, GEE models, ordinal models, clustering, interaction analyses, model diagnostics, sensitivity analyses, and reliability procedures are described. No complex sampling strategy was used. |
| 12c / 14b | Missing data | Methods - Study design and participants / Statistical analysis; Results | All 108 analysed participants had complete outcome data across all four side-condition records (432/432 observations); no imputation was performed. No ultrasonographic measurements existed for the four non-completers. |
| 13a | Participant flow - numbers | Results; Methods - Study design and participants | The enrolled-to-analysed flow is reported: 112 enrolled, four did not complete ultrasonography, and 108 participants/432 side-condition observations were analysed. |
| 13b | Participant flow - reasons | Results; Methods - Study design and participants | The four non-completers were distributed across Eichner A (n=1), B (n=2), and C (n=1). One Eichner C participant could not maintain adequate cooperation; three participants (A n=1, B n=2) chose to discontinue. No ultrasonographic measurements were obtained from them. |
| 13c | Participant flow diagram | Methods - Study design and participants | A complete screened-to-enrolled flow diagram could not be reconstructed because reliable pre-enrollment screening and exclusion counts were unavailable; the complete verifiable enrolled-to-analysed flow is reported in text. |
| 14a, 14c | Descriptive data | Results / Table 1 | Demographic characteristics by Eichner group and the number of participants with available data are reported. |
| 15 | Outcome data | Results / Tables 2-5; Figs. 4-7 | Unadjusted ultrasound-derived outcome summaries, adjusted main-effect and interaction estimates, correlation results, and participant-level bilateral-mean reliability estimates are reported. |
| 16a | Main results | Results / Tables 2-5; Additional file 1: Table S8 | Unadjusted and adjusted estimates, 95% confidence intervals where applicable, and interaction results are reported. The limitations of the exploratory ordinal models are stated in the main Results, and full ordinal and threshold-specific outputs are provided in Additional file 1: Table S8. |
| 16b | Category boundaries | Methods - Participant grouping / Statistical analysis | Eichner groups A, B, and C and their posterior-support definitions are stated; ordered-model coding (A < B < C) is described. |
| 16c | Relative-to-absolute estimates | Not applicable | The study did not estimate risk ratios or other relative risks requiring translation into absolute risk. |
| 17 | Other analyses | Additional file 1 | Secondary sex-stratified analyses, age correlations, post hoc comparisons, correlation matrices, robust regression, model sensitivity analyses, and Eichner-subclass summaries are reported. |
| 18 | Key results | Discussion | Principal findings are summarized with reference to the study objective. |
| 19 | Limitations | Discussion | Cross-sectional inference, incomplete pre-enrollment screening counts, small-number post-enrollment attrition, Eichner-group heterogeneity, protocol-specific measurements, acquisition/pixel-scale issues, reliability scope, and unmeasured covariates are discussed. |
| 20 | Interpretation | Discussion / Conclusion | Interpretation is balanced with biological plausibility, model sensitivity, and methodological caution. |
| 21 | Generalisability | Discussion | The single-center cohort and need for acquisition-standardized, longitudinal, and prosthetic-rehabilitation studies are described. |
| 22 | Funding | Declarations | The funding statement is provided. |
